# Structural insights into apoptotic regulation of human Bfk as a novel Bcl-2 family member

**DOI:** 10.1016/j.csbj.2022.01.023

**Published:** 2022-01-28

**Authors:** Dong Man Jang, Eun Kyung Oh, Hyunggu Hahn, Hyoun Sook Kim, Byung Woo Han

**Affiliations:** a Research Institute of Pharmaceutical Sciences, College of Pharmacy, Seoul National University, Seoul 08826, Republic of Korea; b Research Institute, National Cancer Center, Goyang, Gyeonggi 10408, Republic of Korea

**Keywords:** Bcl-2 family, Bfk, BH domain, Bid, Apoptosis, Crystal structure

## Abstract

Bcl-2 family kin (Bfk), also known as Bcl-2-like 15, plays an essential role in regulating apoptosis by eliciting weak pro-apoptotic responses in the gastrointestinal tract. Human Bfk is a novel Bcl-2 family protein owing to its unique domain composition involving BH2 and BH3. However, the molecular mechanism underlying the regulation of apoptosis by Bfk remains unclear. Here, we first report the crystal structure of human full-length Bfk. Surprisingly, the structure of Bfk adopts a canonical Bcl-2 fold but lacks the hydrophobic cleft, which could accommodate a BH3 domain from other Bcl-2 family proteins. Our biophysical interaction analysis proved that the full-length Bfk itself does not interact with multi-domain Bcl-2 family proteins or a BH3-containing peptide. Instead, Bfk is structurally and functionally reminiscent of Bid, a BH3-only protein in the Bcl-2 family, with similar conformations of helices α3-α5 and the specific motif in helix α5. Not only structural analyses of the full-length Bfk but also molecular dynamics simulation suggested that Bfk elicits its pro-apoptotic activity through a Bid-like apoptotic mechanism in which the BH3 domain is released upon caspase-mediated cleavage and a conformational change of the truncated form. Indeed, the BH3 peptide derived from Bfk exhibited *in vitro* interactions with Bcl-2, Bcl-X_L_, and Bak. These findings provide new insights into the molecular characteristics of Bfk and a valuable foundation for development of a new therapeutic target to control apoptosis.

## Graphical abstract

**Figure:**
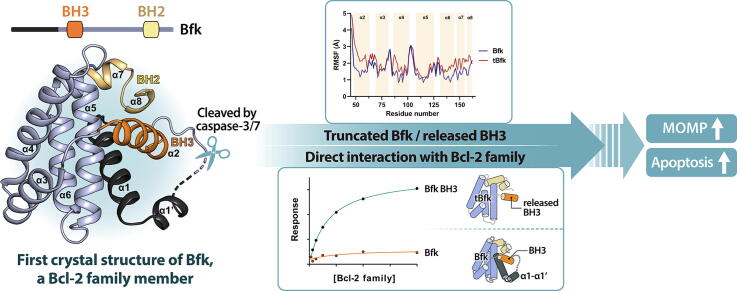

## 1 Introduction

Proteins of the B cell lymphoma-2 (Bcl-2) family are involved in the intrinsic apoptotic pathway that releases cytochrome C into the cytoplasm by regulating mitochondrial outer membrane permeabilization (MOMP) [1], [2]. Bcl-2 family proteins harbor up to four highly conserved regions called Bcl-2 homology (BH) domains, although overall sequence identities of Bcl-2 family proteins are low [3], [4]. Among the four BH domains, a BH3 domain is present in all Bcl-2 family proteins and governs interactions with the hydrophobic cleft of other Bcl-2 family proteins, which is also known as a BH3 and C-terminus binding groove (BC groove) [5].

**Fig. 1:**
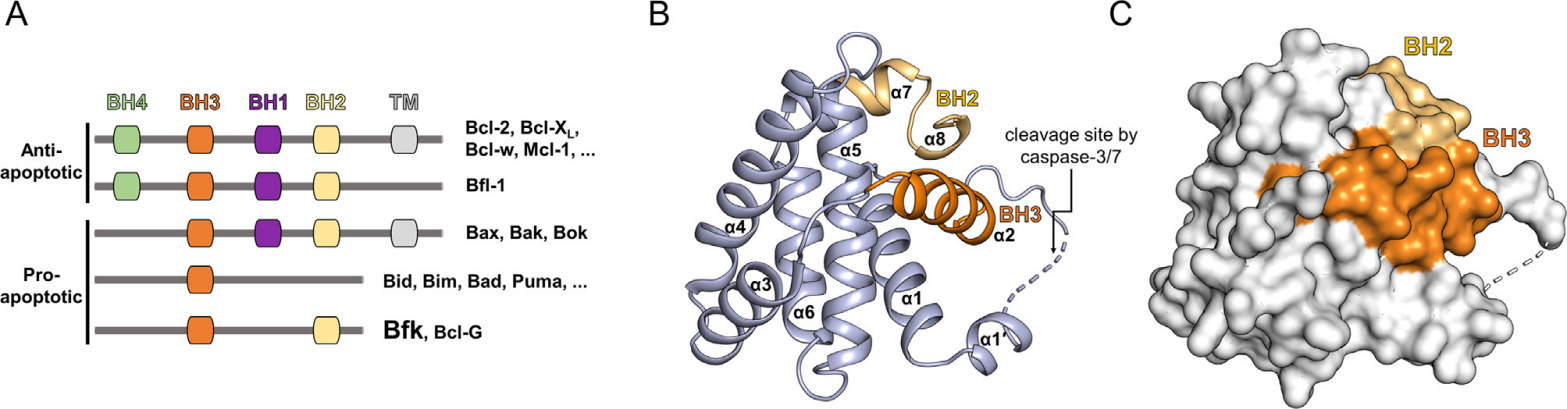
Classification of Bcl-2 family proteins and the overall structure of human full-length Bfk. (A) Bcl-2 family proteins are classified based on their primary functions in apoptosis and Bcl-2 homology (BH) domain compositions. BH domains are depicted by colored boxes. Actual length of proteins and location of BH domains are not considered in the diagram. (B, C) Overall structure of Bfk is displayed as cartoon representation colored in light blue (B) and as surface representation colored in white (C). BH2 and BH3 domains of Bfk are colored in yellow and orange, respectively. The structurally unresolved α1′-α2 loop is presented as a dotted line.

Bcl-2 family proteins are generally classified as either anti-apoptotic (Bcl-2, Bcl-W, Bcl-X_L_, Mcl-1, and Bfl-1) or pro-apoptotic based on their primary functions and BH-domain compositions (Fig. 1a) [3], [6]. The pro-apoptotic proteins can be further sub-divided into pro-apoptotic pore-formers (Bax, Bak, and Bok) or pro-apoptotic BH3-only proteins (Bid, Bim, Bik, Bad, Bmf, Puma, Noxa, and Hrk among others) [6]. The anti-apoptotic proteins and pro-apoptotic pore-formers are multi-domain Bcl-2 family proteins that contain four (BH1–4) or three (BH1–3) domains, respectively, with or without a C-terminal transmembrane domain [1], [7]. The multi-domain Bcl-2 family proteins adopt a structurally conserved Bcl-2 fold comprising two central hydrophobic α-helices and six or seven surrounding amphipathic α-helices [2], [6]. Anti-apoptotic proteins inhibit the activation of pro-apoptotic pore-formers by sequestering the BH3 domains of pro-apoptotic proteins in their BC grooves with high affinity [8], [9]. Otherwise, pro-apoptotic pore-formers undergo structural rearrangements upon the release from anti-apoptotic proteins or the transient binding of BH3-only proteins, which result in oligomerization and pore formation within the mitochondrial outer membrane [10]. Recent mutation studies on Bak, a pro-apoptotic pore-former, showed that the BH1 and BH3 domains play important roles in the activation and oligomerization of Bak and further MOMP [11].

In contrast to multi-domain Bcl-2 family proteins, pro-apoptotic BH3-only proteins are intrinsically disordered and, except for Bid, exhibit pro-apoptotic activity without forming the Bcl-2 fold [12]. Bid is a BH3-only protein known to induce the activation of pro-apoptotic pore-formers [11], [13], although it remains debated whether the direct binding of BH3-only proteins to Bax/Bak is essential to Bax/Bak activation or whether they play an indirect role in the activation by neutralizing only anti-apoptotic Bcl-2 proteins, such as Bcl-X_L_ or Mcl-1 [14]. Full-length Bid maintains the Bcl-2 fold in the cytosol until it is cleaved by caspases [15], [16]. The cleavage of Bid (p22) releases an N-terminal fragment (p7) upon contact with a hydrophobic membrane, whereas the truncated Bid (tBid; p15) undergoes a conformational change to embed it in the mitochondrial outer membrane for further recruitment and activation of Bcl-2 family pore-formers [17], [18]. Recently, tBid is also suggested to mediate MOMP even in absence of Bax and Bak [19].

Some proteins in the Bcl-2 family do not meet the general classification criteria described previously herein owing to their ambiguous functions and compositions of BH domains. Bcl-2 family kin (Bfk) is a novel Bcl-2 family protein that harbors a unique combination of BH2 and BH3 domains [20]. Human *BFK* is predominantly expressed in the gastrointestinal tract, and its enforced expression weakly promotes apoptosis [20]. Additionally, *BFK* expression is significantly downregulated in tumors isolated from various gastrointestinal tissues [21], [22]. A truncated form of Bfk (tBfk), following cleavage of an N-terminal fragment by caspases, elicits stronger pro-apoptotic activity than full-length Bfk [21]. Moreover, tBfk induces apoptosis in a Bax/Bak-dependent manner, whereas co-expression with Bcl-X_L_ and Bcl-W reverses the tBfk-mediated apoptotic activity [23]. Despite the biological significance of Bfk in the onset of apoptosis, the structural and molecular mechanisms underlying apoptotic regulation by Bfk remain elusive.

Here, we report the first crystal structure of human Bfk at 2.70 Å resolution, as a representative of Bcl-2 family proteins comprising a novel combination of BH2 and BH3 domains. The overall structure of Bfk adopts the Bcl-2 fold, similar to those of multi-domain Bcl-2 family proteins. However, structural, biophysical, and computational analyses of Bfk revealed significant similarities with Bid, a BH3-only protein, thus implying that Bfk induces apoptosis activation by adopting the BH3-releasing conformation. These findings provide insights into the apoptotic mechanism of action of Bfk and a valuable foundation for the development of a new therapeutic target to regulate apoptosis.

## 2 Materials and methods

### 2.1 Cloning, expression, and purification of Bfk

A full-length human *BFK* (residues 1–163) encoded from the *BCL2L15* gene was amplified using polymerase chain reaction and cloned into the expression vector pET-28a(+) (Novagen, Madison, WI, USA) between *Nde Ⅰ* and *Xho Ⅰ* restriction sites to contain an N-terminal hexahistidine (His_6_) tag (MGSSHHHHHHSSGLVPRGSH). The *BFK* cloned plasmid was transformed into OverExpress^TM^ C43(DE3) (Lucigen, Middleton, WI, USA), an *Escherichia coli* strain. The transformed cells were cultured in Lysogenic Broth media containing 30 μg/mL kanamycin at 37℃ until OD_600_ reached 0.6, and were added of 0.5 mM isopropyl β-*D*-1-thiogalactopyranoside (IPTG) to induce overexpression. After incubation for an additional 16 h at 20℃, the cells were harvested by centrifugation at 6,000 × g for 10 min. The pelleted cells were resuspended in buffer A (500 mM NaCl, 35 mM imidazole, and 20 mM Tris-HCl at pH 7.5) containing 1 mM phenylmethanesulfonylfluoride and lysed by sonication. The lysate was centrifuged at 35,000 × g for 60 min at 4℃. The supernatant was filtered through a 0.45 μm syringe filter device (Sartorius, Göttingen, Germany) to remove cell debris and any precipitated proteins. For affinity chromatography, the filtrate was applied onto 5 mL HiTrap chelating HP column (GE Healthcare, Chicago, IL, USA) which had been pre-charged with Ni^2+^ and equilibrated with buffer A. The retained proteins were eluted with a gradient of buffer B (500 mM NaCl, 1 M imidazole, and 20 mM Tris-HCl at pH 7.5). For size exclusion chromatography, the eluent was applied onto a HiLoad 16/600 Superdex 75 pg column (GE Healthcare, Chicago, IL, USA) which had been equilibrated with buffer C (200 mM NaCl and 25 mM MES-NaOH at pH 6.0). The purified Bfk was concentrated to 15 mg mL^−1^ using an Amicon Ultra-15 Centrifugal Filter Unit (Merck Millipore, Darmstadt, Germany).

For selenomethionine (SeMet)-derived proteins, the recombinant Bfk proteins were overexpressed in *E. coli* strain B834(DE3) using the media containing M9 minimal salts (Sigma-Aldrich, Darmstadt, Germany) and amino acid mix containing *L*-selenomethionine (Sigma-Aldrich, Darmstadt, Germany). The protein was expressed and purified as for the native Bfk.

### 2.2 Crystallization, data collection, and structure determination

Purified Bfk proteins were crystallized at 22℃ using the sitting-drop vapor diffusion method by mixing 0.5 μL proteins and 0.5 μL crystallization solution. Initial crystals were obtained under commercial crystallization screening conditions containing 0.1 M Bis-Tris at pH 7.5 and 25% polyethylene glycol (PEG) 3,350 of the ShotGun 1 kit (Molecular dimension, Cambridge, England). The crystals suitable for data collection were optimized at 16℃ using the micro-seeding method. The micro-seeds of crystals were prepared from the initial crystals of Bfk using Seed Bead^TM^ Kits (Hampton Research, Aliso Viejo, CA, USA) according to the manufacturer’s instructions. The SeMet-derived crystals were obtained in the same manner as native Bfk.

The crystals were cryoprotected in the crystallization solution supplemented with 17% glycerol and flash-frozen in a 100 K nitrogen gas stream for exposure to X-ray beams. The X-ray diffraction data for native Bfk was collected to 2.70 Å using an Eiger 9 M detector system (Dectris Ltd., Baden, Switzerland) at beamline 5C experimental station at Pohang Light Source, Korea. To solve the phase problem, single-wavelength anomalous diffraction (SAD) data of SeMet-derived Bfk was collected to 2.45 Å at the same beamline. The raw data were indexed and scaled using the *HKL*2000 program suite [24] and *XDS* [25]. SAD phases were calculated with *Autosol* in the *PHENIX* software suite [26], and further improved by density modification using the automatic model building program *Resolve* [27]. Then, the model was used as a template for molecular replacement method using *MolRep* [28] to obtain phases for the diffraction data collected from the native Bfk crystals. The resulting models were further refined using iterative cycles of model building with *Coot* [29] and *Refmac5* [30] in *CCP4i* program suite [31]. Since the diffraction data of the Bfk native crystal was analyzed as having twin fractions by *Xtriage* of the *PHENIX* software suite, which caused a subsequent problem with abnormally high R-factors, the intensity-based twin refinement of *Refmac5* was applied. After the refinement with twin fractions of 0.543 and 0.457 for the twin operators of (h, k, l) and (-h, -k, l), respectively, the R-factors and quality of the electron density were suitably improved. All refinement steps were checked using an *R*_free_ value [32] calculated for a randomly chosen 5% of reflections and the reliability of refined models was validated using *MolProbity* [33] and the Research Collaboratory for Structural Bioinformatics (RCSB) Protein Data Bank (PDB) validation server. The statistics of data collection and refinement are summarized in Table 1.

**Table 1:** Statistics for the data collection and model refinement.

	SeMet-derived Bfk	Native Bfk
**A. Data collection**		
X-ray source	PLS-5C	PLS-5C
X-ray wavelength (Å)	0.97918	0.97957
Data process suite	*HKL*2000	*XDS*
Space group	*P*3_2_	*P*3_2_
a, b, c (Å)	116.16, 116.16, 27.68	116.50, 116.50, 27.63
α, β, γ (⁰)	90.00, 90.00, 120.00	90.00, 90.00, 120.00
Resolution range^a^ (Å)	50.00–2.45 (2.49–2.45)	30.00–2.70 (2.77–2.70)
Total/unique reflections	47,845/15,406^c^	40,763/11,459
Redundancy^a^	3.1 (2.0)^c^	3.6 (3.5)
Completeness (%)^a^	99.1 (90.9)^c^	99.3 (98.1)
<I/σ_I_ >^a^	12.32 (1.06)^c^	13.5 (1.96)
*R_sym_* (%)a,^b^	7.1 (78.4)^c^	7.7 (80.9)
CC_1/2_^a^	0.997 (0.803)^c^	0.998 (0.599)
**B. Model refinement**		
PDB code	7CCM	7CCL
Resolution range (Å)	50.00–2.45	30.00–2.70
*R_work_* / *R_free_*^d^ (%)	21.2/25.4	15.8/19.0
Twin fraction^e^		0.543, 0.457
R.m.s. deviations		
Bond lengths (Å)	0.0089	0.0050
Bond angles (⁰)	1.1194	1.1671
Ramachandran^f^		
Favored / Allowed (%)	98.82/1.18	99.76/0.24
Outliers (%)	0.00	0.00
Rotamers^f^		
Favored / Allowed (%)	98.31/1.69	99.15/0.85
Outliers (%)	0.00	0.00
No. of non-hydrogen atoms		
Protein	3,310	3,306
water	19	11
Average B factor (Å^2^)		
Protein	42.81	70.67
Water	34.54	44.41

a Values in parentheses refer to the highest resolution shell.

b *R_sym_* = Σ_h_Σ_i_|I(h)_i_–<I(h)>|/Σ_h_Σ_i_I(h)_i_, where I(h) is the intensity of reflection h, Σ_h_ is the sum over all reflections, and Σ_i_ is the sum over i measurements of reflection h.

c Friedel pairs were treated as separate observations.

d *R_free_* = Σ||*F*_obs_|–|*F*_calc_||/Σ|*F*_obs_|, where *R_free_* is calculated for a randomly chosen 5% of reflections that were not used for structure refinement. *R_work_* is calculated for the remaining reflections.

e Twin fractions for the twin operators of (h, k, l) and (-h, -k, l), in order, are calculated during the intensity-based twin refinement of *Refmac5* in the CCP4i suite. ^f^ Values obtained using *MolProbity*.

### 2.3 Protein preparations for Bcl-2, Bcl-X_L_, and Bak

The genes, encoding Bcl-2 (residues 1–207), Bcl-X_L_ (residues 1–209), and Bak (residues 23–186), were each cloned into the expression vector pET-28a(+) (Novagen) between *Nde Ⅰ* and *Xho Ⅰ* restriction sites to contain the N-terminal His_6_ tag. The cloned plasmids of Bcl-2, Bcl-X_L_, and Bak were each transformed into *E. coli* Rosetta^TM^ 2(DE3), Rosetta^TM^ 2(DE3), and Rosetta^TM^ 2(DE3)pLysS strains, respectively. Bcl-2- or Bcl-X_L_ transformed cells were cultured in Lysogenic Broth media containing 30 μg/mL kanamycin at 37℃ until OD_600_ reached 0.6. Overexpression of the cells were induced by adding 0.5 mM IPTG to induce overexpression, followed by further incubation for additional 16 h at 20℃. Bak-transformed cells were identically cultured in Terrific Broth media. The purification steps were identically performed as described above. The size exclusion chromatography was performed using buffer D (150 mM NaCl, 20 mM Tris at pH 7.5, and 0.5 mM TCEP). The proteins of Bcl-2, Bcl-X_L_, and Bak were buffer-exchanged to the HBS-EP buffer containing 10 mM HEPES at pH 7.5, 150 mM NaCl, 0.05 mM ethylenediaminetetraacetic acid (EDTA), and 0.005% Tween 20 for further analyses.

### 2.4 Surface plasmon resonance analyses

The binding affinities of the synthetic BH3 peptide derived from Bfk (residues 47–67; SFDVAIIAGRLRMLGDQFNGE) or the full-length Bfk with Bcl-X_L_, Bcl-2, and Bak were investigated by surface plasmon resonance (SPR) kinetics experiments using the Biacore T200 apparatus (GE Healthcare, Chicago, IL, USA). The Bfk BH3 peptide and the full-length Bfk prepared in 10 mM sodium acetate buffer (at pH 5.0 and pH 3.6, respectively) were immobilized on a CM5 sensor chip with the HBS-EP buffer using the amine coupling kit containing 0.1 M *N*-hydroxysuccinimide and 0.4 M 1-ethyl-3-(3-dimethylaminopropyl) carbodiimide hydrochloride according to the manufacturer’s protocol (GE Healthcare). The remaining activated carboxyl groups on the CM5 sensor chip were deactivated with 1 M ethanolamine at pH 8.5. Reference flow cells were treated identically without the ligands. The responses for the reference flow cells were subtracted from each sample. Bcl-2 and Bcl-X_L_ at concentrations of 3.13, 6.25, 12.5, 25.0, 50.0, 100, and 200 nM, and Bak at concentrations of 1.25, 2.50, 5.00, 10.0, 20.0, and 40.0 μM were injected over the chip at a rate of 30 μL min^−1^ for 90 s, followed by dissociation for 300 s in multi-cycle reactions. The sensor chip was regenerated using 5 mM NaOH for 10 s between cycles. Biacore T200 evaluation software (GE Healthcare, Chicago, IL, USA) was used to calculate the kinetics data using the 1:1 binding model.

The binding affinities of the synthetic BH3 peptide derived from Bid (residues 79–100; EDIIRNIARHLAQVGDSMDRS) as a representative BH3 peptide with Bfk, Bcl-X_L_, Bcl-2, and Bak were investigated by SPR affinity experiments using the Biacore T200 apparatus (GE Healthcare, Chicago, IL, USA). The Bid BH3 peptide in 10 mM sodium acetate at pH 4.0 was immobilized on a CM5 sensor chip as described above. Reference flow cells were treated identically without the ligands. The responses for the reference flow cells were subtracted from each sample. Bcl-2 and Bcl-X_L_ at concentrations of 3.13, 6.25, 12.5, 25.0, 50.0, 100, and 200 nM, and Bak at concentrations of 1.25, 2.50, 5.00, 10.0, 20.0, and 40.0 μM were injected over the chip at a rate of 30 μL min^−1^ for 90 s, followed by dissociation for 300 s in multi-cycle reactions. The sensor chip was regenerated using 5 mM NaOH for 10 s between cycles. Biacore T200 evaluation software (GE Healthcare, Chicago, IL, USA) was used to calculate the kinetics data using the 1:1 binding model.

### 2.5 Molecular dynamics simulation

Protein models for full-length or truncated forms of Bfk and Bid are prepared using *Protein Preparation Wizard* [34] in *Schrödinger* software with OPLS4 force field. In particular, an unmodelled loop in the crystal structure of Bfk were generated by *Prime* [35] in *Schrödinger* software. All molecular dynamics (MD) simulations were performed using the *Desmond* [36] in *Schrödinger* software. Periodic boundary conditions using orthorhombic boxes buffered at 10 × 10 × 10 Å or 15 × 15 × 15 Å distances were applied for explicit solvent simulations. The system was solvated with water adopting TIP3P water model and 150 mM NaCl after it was neutralized with sodium (or chloride) ions to electrically balance the system. The solvated system containing protein was energy-minimized and relaxed for 100 ps by the minimization step of *Desmond* using OPLS2005 force field. MD simulations were conducted in the NPT (isothermal and isobaric simulation) ensemble, where Martyna‐Tobias‐Klein method [37] and Nose‐Hoover thermostat algorithm [38] were used for isotropic pressure (1 atm) and constant temperature (300 K), respectively. Total 100–300 ns simulations were run and saved as trajectories at 100–300 ps intervals with two independent replicates. The trajectories were analyzed using *Simulation Interaction Diagram* in *Desmond* and *VMD* (Visual Molecular Dynamics) 1.9.3 [39].

### 2.6 Data deposition

The coordinates and structure factors of SeMet-derived and native Bfk are available in the Protein Data Bank under accession codes 7CCM and 7CCL, respectively.

## 3 Results

### 3.1 The overall structure of human Bfk adopts the Bcl-2 fold of multi-domain Bcl-2 family proteins

Bfk has a unique domain composition involving only BH2 and BH3 domains compared with multi-domain Bcl-2 family proteins that harbor three (BH1–3) or four (BH1–4) domains with or without a C-terminal transmembrane helix (Fig. 1a). To gain insight into the molecular basis for the function of human Bfk, we determined the crystal structure of human full-length Bfk at 2.70 Å resolution using a single-wavelength anomalous dispersion method with selenomethionine-substituted crystal of Bfk (Table 1). Since overall structures and positions of key residues of native and selenomethionine-derived Bfk are nearly identical (a Cα root-mean-square deviation value of 0.184), hereinafter we will discuss the features of the native Bfk structure for clarity (Table 1). Interpretation for the native structure with the relatively high B-factor (an average value of 70.7 Å^2^), probably due to the crystal quality and intrinsically flexible nature of its fold, was complemented and confirmed by comparing with the selenomethionine-derived structure (an average protein B-factor of 42.8 Å^2^). The Bfk structure consists of two central hairpin-shaped hydrophobic α-helices (α5–α6) encompassed by six amphipathic α-helices (α1–α2, α3–α4, and α7–α8) (Fig. 1b). The overall structure of Bfk adopted the Bcl-2 fold typical of multi-domain Bcl-2 family proteins, which is consistent with the results of structural similarity analysis using the *Dali* server [40] where the overall structure of Bfk resembles that of Bcl-X_L_ (Z-score of 11.6; PDB code 6RNU), Mcl-1 (Z-score of 11.4; PDB code 6QGD), Bcl-2 (Z-score of 10.3; PDB code 6QGH), Bak (Z-score of 8.4; PDB code 2M5B), and Bax (Z-score of 7.8; PDB code 5 W60) (Supplementary Fig. S1). However, there were subtle differences from the other structures; for example, Bfk contains an additional alpha helix (α1′) following helix α1 and a helix α8 shorter than those of the multi-domain proteins (Fig. 1b and Supplementary Fig. S1). Furthermore, noticeable structural differences were found among them in the conformations of helices α2–α4 (Fig. 1b,c and Supplementary Fig. S1), as described later in detail.

### 3.2 The Bfk structure lacks the typical BC groove, albeit with the Bcl-2 fold

**Fig. 2:**
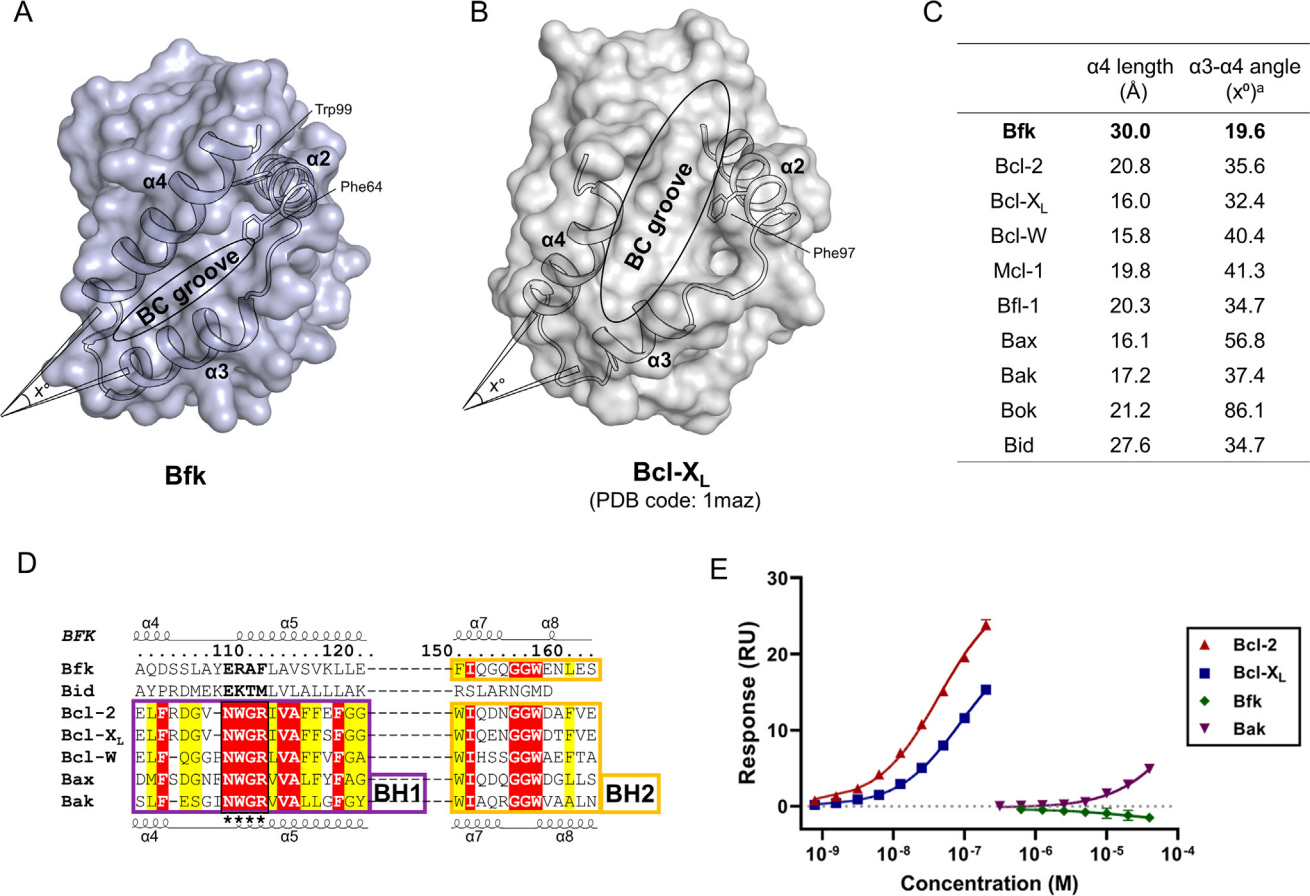
The comparison of Bfk and multi-domain Bcl-2 family proteins. (A, B) Overall structures of Bfk (A) and Bcl-X_L_ (B) are displayed as surface representations colored in light blue and white, respectively. Cartoon representations for helices α2-α4 are depicted with black outlines on the surface representations. A few residues on helices α2-α4 are drawn as stick representations colored in black. BC grooves in the center of helices α2-α4 are indicated with black circles. Angles (x°) of helices α3-α4 are arbitrarily drawn. (C) Simplified measurements of BC grooves. The structures of Bcl-2 (PDB ID: 1G5M), Bcl-X_L_ (1MAZ), Bcl-W (1O0L), Mcl-1 (2MHS), Bfl-1 (5WHI), Bax (1F16), Bak (2YV6), Bok (6CKV), and Bid (2BID) are used for a calculation. The length of helix α4 and angle between helices α3-α4 are measured by a PyMOL plugin, AngleBetweenHelices. (D) Sequence alignment of Bfk with Bid and multi-domain Bcl-2 family proteins. Identical and highly conserved residues are colored in red and yellow, respectively. BH1 and BH2 domains are indicated by boxes colored in magenta and yellow, respectively. Secondary structures and atom numbers of Bfk are shown above the alignment, and secondary structures of multi-domain Bcl-2 family proteins are shown below. Asterisks indicate the NWGR motif of BH1 domain. (E) Affinity analysis of a representative BH3 peptide (EDIIRNIARHLAQVGDSMDRS) against Bfk and multi-domain Bcl-2 family proteins by a surface plasmon resonance (SPR) method. Responses to an immobilized BH3 peptide are plotted against various concentrations of Bcl-2, Bcl-X_L_, Bak, and Bfk. Values represent means ± standard deviation (SD) of two independent experiments.

Multi-domain Bcl-2 family proteins typically form a large hydrophobic cleft, also known as the BC groove, surrounded by helices α2–α5 (Fig. 2b), which enables their direct interaction with the BH3 domain of other Bcl-2 family proteins to regulate intrinsic apoptotic signals [5]. The Bfk structure with the canonical Bcl-2 fold was found to possess helices α2–α5 at similar positions as shown in multi-domain proteins, but lacked the BC groove owing to a distinct conformation of helices α3–α4, with a small angle between them and a long helix α4 of up to 20 residues (Fig. 2a). In the structure of Bfk, helices α3–α4 with an angle much smaller than those in the multi-domain proteins cannot make a large cleft between them, and the helix α4 that is long enough to reach helix α2 blocks a groove extension via hydrophobic contact between Trp99 on helix α4 and Phe64 on the α2-α3 loop with a distance of 3.5 Å (Fig. 2a). Interestingly, the helix α2 is positioned to lock off the top of the BC groove, which completely blocks the P3 and P4 among four pocket regions (P1–P4) present in the typical BC groove of other Bcl-2 family proteins (Supplementary Fig. S2). In contrast, the structure of Bcl-X_L_, a representative multi-domain Bcl-2 family protein, has a large angle between helices α3-α4 and a short helix α4, thereby forming a large BC groove that harbors P1–P4 pockets responsible for hydrophobic contacts with other BH3 domain [41], [42] (Fig. 2b and Supplementary Fig. S2).

When we measured the angle (x°) between helices α3-α4 and the length (Å) of helix α4 using structures of Bcl-2 family proteins in the BH3-unbound state, the differences between Bfk and other Bcl-2 family proteins were clearly compared (Fig. 2c and Supplementary Fig. S3). The structure of Bfk exhibited a remarkably small angle between helices α3-α4 and a longer helix α4 (19.6° and 30.0 Å, respectively), whereas the structures of multi-domain Bcl-2 family proteins, including Bcl-2, Bcl-X_L_, Bcl-W, Mcl-1, Bfl-1, Bax, Bak, and Bok, show large angles between helices α3-α4 (>32.4°) and a shorter helix α4 (15.8–20.9 Å) (Fig. 2c). These findings indicate that the Bfk structure, albeit with the Bcl-2 fold, does not bear the BC groove to accommodate a BH3 domain from other Bcl-2 family proteins.

In addition, owing to the absence of a BH1 domain, Bfk also lacks a NWGR motif that in general is conserved in multi-domain Bcl-2 family proteins (Fig. 2d), which is known to play a key role in the recognition of a BH3 domain from other Bcl-2 family proteins [43]. This led us to speculate that owing to the absence of both the BC groove and the NWGR motif, Bfk cannot recognize a BH3 domain of other Bcl-2 family proteins. In support of this speculation, using a surface plasmon resonance (SPR) method, we measured the binding ability of the BH3 domain from Bid as a representative BH3 peptide to Bfk in comparison with that of other Bcl-2 family proteins such as Bcl-X_L_, Bcl-2, and Bak. Bfk showed no appreciable affinity for the BH3 peptide at concentrations up to 40 μM, whereas Bcl-X_L_, Bcl-2, and Bak expectedly had binding affinity with dissociation constant (*K*_D_) values of 87 (±1) nM, 44 (±4) nM, and 37 (±14) μM, respectively (Fig. 2e and Supplementary Fig. S4). Therefore, unlike other multi-domain Bcl-2 family proteins, full-length Bfk does not possess the NWGR motif and the BC groove suitable for the recognition of a BH3 domain from other Bcl-2 family proteins, implying that the full-length form of Bfk remains inert with respect to apoptotic regulation.

### 3.3 The α1′-α2 loop, cleavable by caspases, is anchored to a novel pocket on the other Bfk molecule

**Fig. 3:**
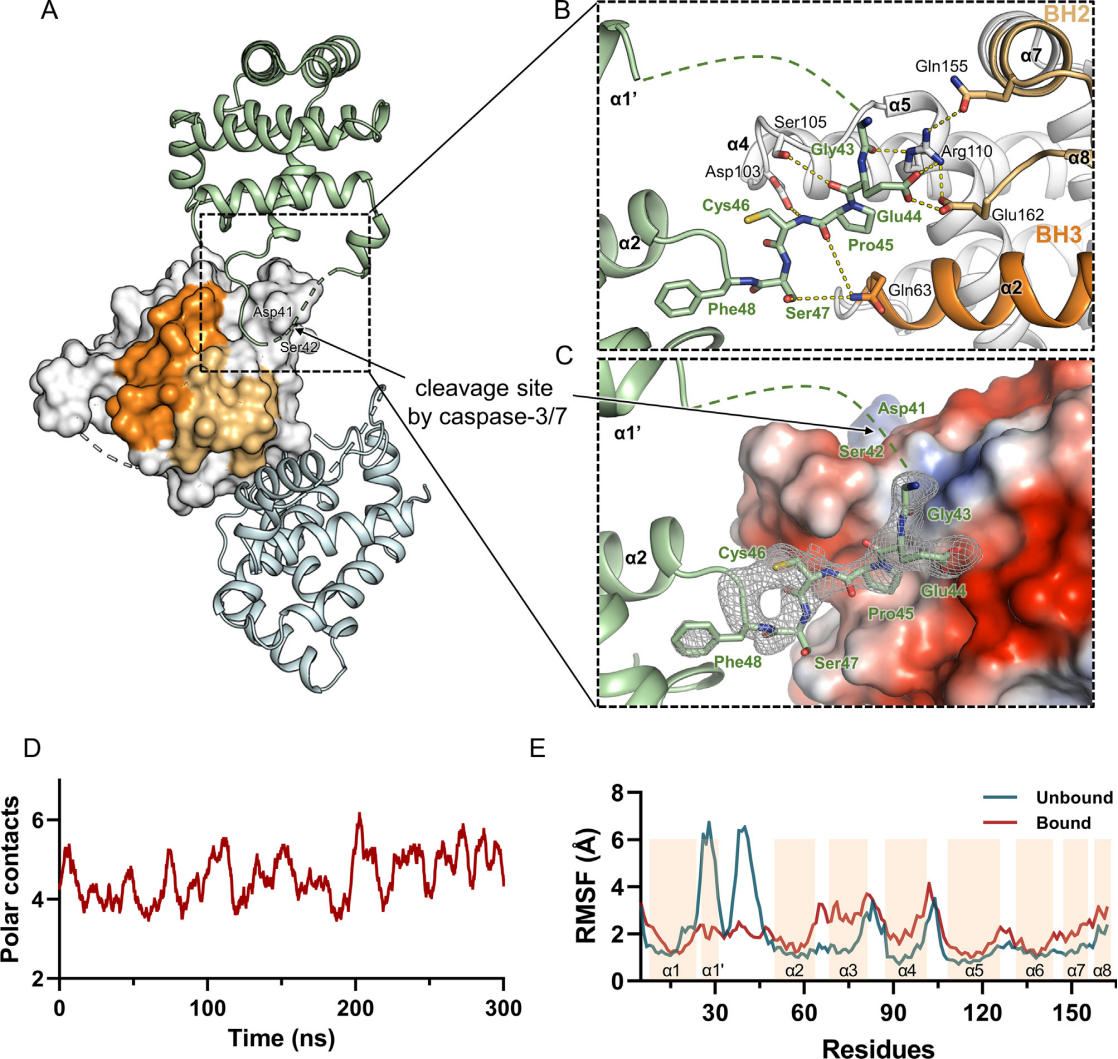
Interaction networks between inter-molecules of Bfk. (A) The crystallographic trimer structure of Bfk. One molecule at the center and two adjasent molecules on both sides are shown as surface (white) and cartoon (green and pale-cyan) representations, respectively. BH2 and BH3 domains are colored in yellow and orange, respectively. (B, C) Close-up views of the interface of two Bfk molecules. The secondary structures are depicted as cartoon representations colored in green and white for each of two molecules. BH2 and BH3 domains are colored in yellow and orange, respectively. The residues lined in the interface are shown with stick representations and labeled in green and black for each of two molecules. The hydrophilic interactions including hydrogen bonds and the structurally invisible region are depicted by yellow and green dotted lines, respectively. (C) One molecule of Bfk is displayed as a surface representation generated by vacuum electrostatics, which indicates negative and positive charges by red and blue, respectively. The omit maps (*mFo* – *DFc*, contoured at 2.0 σ) for residues visible in the α1′–α2 loop are displayed with white-colored mesh. (D) The number of polar contacts between two molecules of Bfk over 300 ns molecular dynamics (MD) simulation. Polar contacts between two Bfk molecules within the distance of 5 Å are calculated by VMD v.1.9.4. and depicted as red-colored lines with the smoothing setting by GraphPad PRISM v.9. (E) Root-mean-square fluctuation (RMSF) values for the bound (red) and unbound (green) forms of Bfk during 300 ns simulation are plotted to corresponding residues. Invisible residues in the loop connecting helix α1′ and helix α2 are modeled using Prime in Schrödinger2021-2. The secondary structures for helices are indicated with wheat colored-boxes.

A part (residues 32–42) of the loop between helices α1′ and α2 (α1′-α2 loop; residues Ser31–Asp49) could not be modeled due to the lack of electron densities, presumably reflecting the flexible nature of the region (Fig. 1b). The sequence analysis of Bfk revealed the presence of a cleavage site (Asp41-Ser42 following a DEVD motif) targeted by caspase-3/7 in the α1′-α2 loop region, as evidenced by peptide and proteomic studies for substrate preference motifs of caspases [23], [44]. Notably, in the Bfk crystal structure, the Gly43–Ser47 region of the α1′-α2 loop that is located immediately adjacent to the cleavage site was clearly visible through stabilization via intermolecular interactions among three monomeric molecules in the crystallographic asymmetric unit. That is, the loop region of one molecule was shown to be docked to an uncharacterized pocket on the other molecule, which was mainly formed by helix α2, the α4-α5 loop, helix α5, and helix α8 (Fig. 3a-c). Indeed, the loop residues between helices α1′ and α2 formed extensive interaction networks via several hydrogen bonds and van der Waals contacts in the novel pocket, where the sidechain of Glu44 was determined to form two hydrogen bonds with the sidechains of Glu162 and Arg110 from the other molecule (Fig. 3b). In addition, the mainchains of Glu44, Pro45, and Cys46 and the sidechain of Ser47 made polar interactions with the sidechains of Ser105, Asp103, and Gln63 (Fig. 3b). In the middle of the unknown pocket, Pro45 was found to occupy a hydrophobic region surrounded by helices α2, α4, and α5 from the other molecule (Fig. 3b,c). In molecular dynamics (MD) simulations using two interacting molecules of Bfk, these extensive intermolecular interactions between the loop region and the novel pocket were constantly sustained over a 300 ns simulation (Fig. 3d). Moreover, the comparison of root-mean-square fluctuation (RMSF) values from the MD simulations with one Bfk molecule alone or with two Bfk interacting molecules (Supplementary Fig. S5) indicated that the flexible α1′-α2 loop in the unbound state (the green line in Fig. 3e) appeared to be stabilized when bound to another Bfk molecule (the red line in Fig. 3e), possibly allowing the next cleavage site to be positioned and poised for the attack of caspases. Considering that the formation of truncated Bfk through caspase-mediated cleavage is necessary to become a pro-apoptotic protein [23], the anchoring of the loop region adjacent to the cleavage site to the novel pocket from the other molecule might contribute to Bfk activation for pro-apoptotic activity.

### 3.4 The helix α5 specific for Bfk and Bid contributes to their characteristic Bcl-2 fold formation

**Fig. 4:**
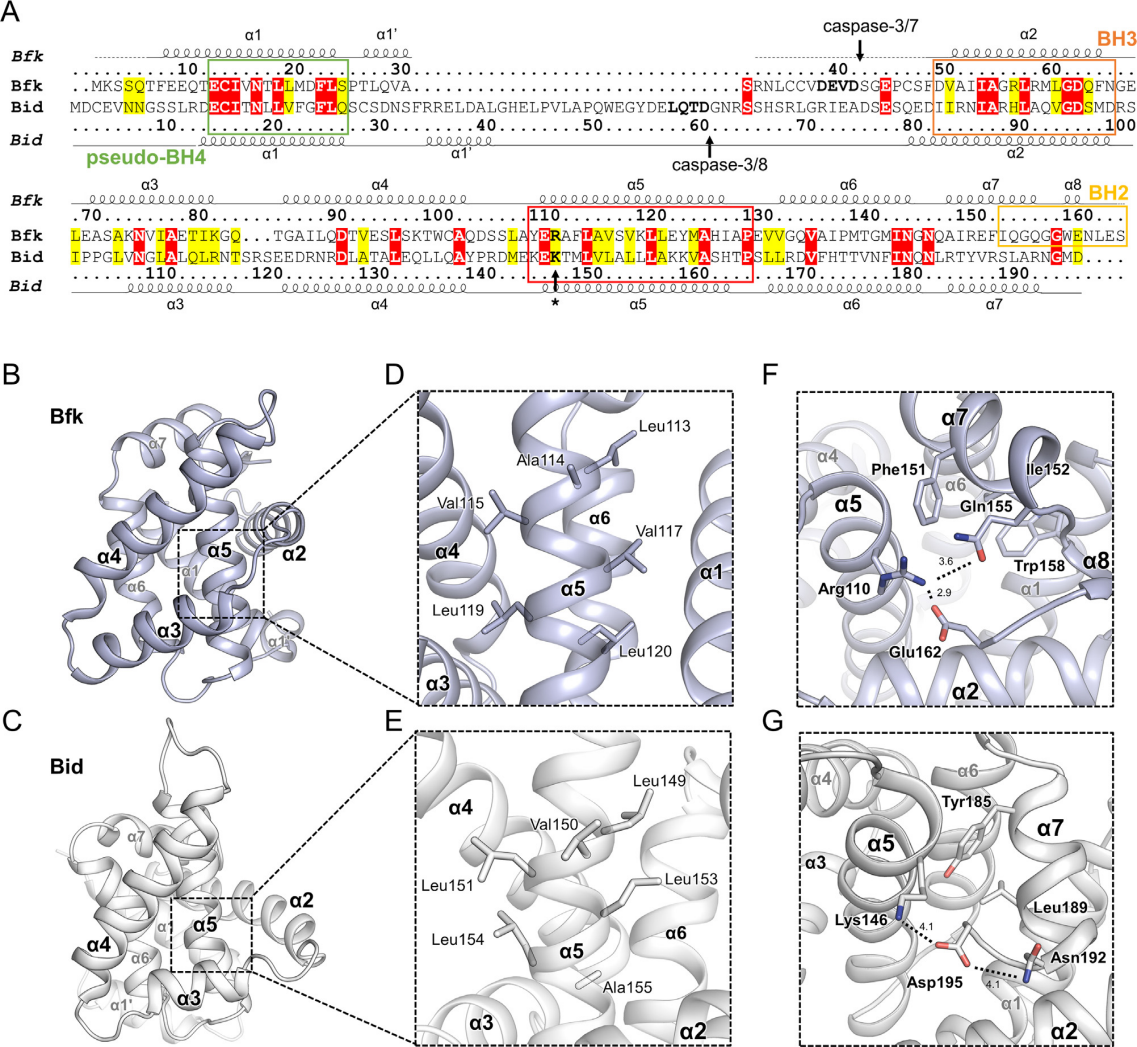
Sequence alignment and structural comparison between Bfk and Bid. (A) Sequence alignment between Bfk and Bid. Identical and highly conserved residues are colored in red and yellow, respectively. Secondary structures and atom numbers of Bfk and Bid are shown above and below their sequences, respectively. Dotted lines of secondary structures indicate unmodeled loops due to lack of electron density. Green-, orange-, yellow-, and red-colored boxes on the sequence alignment indicated pseudo-BH4, BH3, BH2 domains, and the region corresponding BH1 domain, respectively. The arrows with the labels of caspase-3/7 or caspase-3/8 indicate the caspase cleavage site. The arrow with the asterisk indicates the residue corresponding a tryptophan of NWGR motif. (B, C) Overall structures of Bfk and Bid displayed as cartoon representations colored in light blue and white, respectively. (D, E) Close-up views of helix α5 in the structures of Bfk and Bid. Residues on the helix α5 are displayed as stick representations. (F, G) Interaction networks between helix α5 and C-terminal regions in the structures of Bfk and Bid. Secondary structures are shown as cartoon representations and residues participating in the interaction network are depicted with stick representations. Oxygen and nitrogen atoms are colored in red and blue, respectively.

Bid is the only protein that has the helix α4 with a length comparable to that of Bfk among Bcl-2 family proteins (Fig. 2c). In addition, structures of both Bfk and Bid share the unique Bcl-2 fold lacking a canonical BC groove [45] (Supplementary Fig. S1). Bfk has a high sequence similarity (of 52.9% and 17.8% sequence identity) with Bid, showing that the two commonly contain the caspase cleavage sites on the flexible loop before the BH3 domain of helix α2 (Fig. 4a). Moreover, Bfk and Bid have helix α5 with a highly conserved sequence, whereas multi-domain Bcl-2 family proteins instead share a BH1 domain in this region (Fig. 4a,2d). Since the helix α5 comprising the BH1 domain in multi-domain Bcl-2 family proteins plays a central role in forming the solvent accessible hydrophobic BC groove in the Bcl-2 fold [46] (Supplementary Fig. S1), Bfk and Bid, which lack the BH1 domain component, instead possess a characteristic helix α5, forming a Bcl-2 fold distinct from the multi-domain Bcl-2 family proteins. Indeed, when analyzing structures of Bfk and Bid, it was shown that the intrinsic properties of helices α5 of Bfk and Bid influence the conformation of their peripheral helices α3–α4 in a similar way, obstructing the BC groove in both structures (Fig. 4b,c).

In detail, the structures of Bfk and Bid revealed that they share aliphatic amino acids on helix α5 with similar conformations; Leu113, Ala114, Val115, Val117, Leu119, and Leu120 in the Bfk structure corresponded to Leu149, Val150, Leu151, Leu153, Leu154, and Ala155 in the Bid structure, respectively (Fig. 4d,e). In both Bfk and Bid, the helix α5 contains positively charged residues of Arg110 and Lys146 at the N-terminus of the helix, respectively, instead of a tryptophan residue (such as Trp144 of Bcl-2), which is highly conserved in multi-domain Bcl-2 family proteins as one residue of the NWGR motif in the BH1 domain (Fig. 4f,g and Supplementary Fig. S6). As a result, Arg110 of Bfk established hydrophilic interactions with Gln155 and Glu162 of Bfk on the C-terminal α7-α8 helices, and similarly, Lys146 of Bid interacted with Asp195 and Asn192 of Bid (Fig. 4f,g). In contrast, the corresponding tryptophan residue in multi-domain Bcl-2 family proteins makes structurally conserved hydrophobic contacts with a WIxxxGGW motif of BH2 domain at helices α7-α8 (Supplementary Fig. S6a), which is essential for the Bcl-2 family heterodimerization-mediated regulation of apoptosis [46]. In order to investigate a structural impact of the distinct residue in the helix α5 of Bfk and Bid, we conducted a MD simulation using mutant structures (Bfk R110A, Bfk R110W, Bid K146A, and Bid K146W) and observed that the interaction networks between helix α5 and helices α7-α8 mediated by Arg110 of Bfk or Lys146 of Bid significantly disappeared in the mutant forms (Supplementary Fig. S7). Owing to such characteristics of helix α5, it was suggested that Bfk and Bid form their own core structures that differ from the BC groove-preserving multi-domain Bcl-2 family proteins, probably for further Bid-like action releasing the BH3 domain. Therefore, the full-length structures of Bfk and Bid, at first glance, seem to adopt a fold similar to those of other multi-domain proteins, but they completely differ in the way they form their inner core, thereby remaining inactive and unable to interact with other Bcl-2 family proteins until they are further activated by caspases.

### 3.5 The truncated form of Bfk is suggestive of its active characteristics

**Fig. 5:**
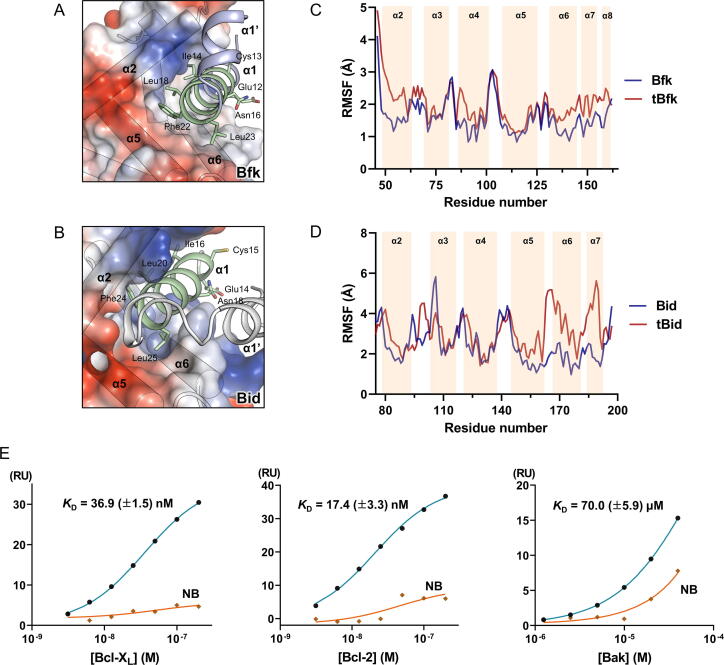
Active characteristics of the truncated forms of Bfk (tBfk) and Bid (tBid). (A, B) N-terminal fragments of Bfk (A) and Bid (B) bound to the core region of the Bcl-2 fold. The core regions representing truncated forms are shown as surface representations gererated by vacuum electrostatics, which indicates negative and positive charges by red and blue, respectively. Secondary structures for helices of the core regions are depicted with cartoon cylindrical helices on the surface representation. The regions for N-terminal fragments are displayed as cartoon representations colored in green for the pseudo-BH4 domain and white for the others. Conserved residues of Bfk and Bid are displayed with stick representations. (C, D) MD simulations of full-length or truncated forms of Bfk (C) and Bid (D). RMSF values for the full-length (blue) and truncated (red) forms during 100 ns simulation are plotted to corresponding residues. Residues ranging from helix α2 to the end are only shown for the comparison. The secondary structures for helices are indicated with wheat colored-boxes. (E) Interaction analyses of the full-length Bfk or the Bfk BH3 peptide for Bcl-X_L_, Bcl-2, and Bak, using a SPR method. Response unit (RU) indicates binding at increasing concentrations (3.13, 6.25, 12.5, 25.0, 50.0, 100, and 200 nM for Bcl-X_L_ and Bcl-2, and 1.25, 2.50, 5.00, 10.0, 20.0, and 40.0 μM for Bak) of Bcl-X_L_, Bcl-2, and Bak to immobilized full-length Bfk (orange) or Bfk BH3 peptide (green). Equilibrium dissociation constant (*K*_D_) values shown on the right side are calculated using a steady-state affinity analysis and represent means ± SD of two independent experiments.

Bfk and Bid have in common the pseudo-BH4 domain containing an ECIxNxLxxxFL sequence (where × represents any amino acid) near the N-terminus (Fig. 4a), which has been known to suppress pro-apoptotic activity until the full-length Bid is cleaved by caspases and becomes a truncated form (tBid) by releasing the N-terminal fragment [15], [16], [45]. In the structures of Bfk and Bid, the N-terminal regions containing the pseudo-BH4 domain were found to have an additional helix α1′ following helix α1, although their orientations were different (Fig. 5a,b). The helices α1 and α1′ were shown to cover the core region of the Bcl-2 fold; Ile14, Ile18, and Phe22 on helix α1 of Bfk (equivalent to Ile16, Leu20, and Phe24 of Bid) established hydrophobic contacts with the groove formed by helices α2, α5, and α6 (Fig. 5a,b). Moreover, the conserved residues Glu12, Cys13, and Asn16 of Bfk (equivalent to Glu14, Cys15, and Asn18 of Bid) on the opposite side of helix α1 were equally exposed to solvent, which was determined to contribute to similar features of the surface formed by helix α1 (Fig. 5a,b). Considering that the helices α1 and α1′ are bound to the core structure in full-length Bfk and Bid, as proposed in tBid formation [17], [18], the detachment of the helices should be preceded after caspase cleavage, thus exposing and releasing the helix α2 containing the BH3 domain that is actually involved in the pro-apoptotic activity of Bfk.

To determine whether the flexibility of helix α2 in the truncated form could be increased compared with that of the full-length form, we performed MD simulations using full-length and truncated structures of Bfk and Bid. A comparison of RMSF values between them showed that the helix α2 became more flexible in the truncated forms of both Bfk and Bid (Fig. 5c,d), implying that the helix α2 constituting the BH3 domain can be easily released for the interaction with other Bcl-2 family proteins. Since we could not obtain a stable truncated Bfk (tBfk) in aqueous solution, we measured the binding affinity of the Bfk BH3 peptide (residues 47–67; SFDVAIIAGRLRMLGDQFNGE) instead of that of tBfk or full-length Bfk versus those of other Bcl-2 family proteins to confirm that the released BH3 domain can bind to anti-apoptotic Bcl-2 proteins or pro-apoptotic pore-formers. Indeed, whereas the full-length Bfk exhibited no binding affinity for Bcl-2, Bcl-X_L_, or Bak (the orange lines in Fig. 5e), the Bfk BH3 peptide showed strong binding affinity for Bcl-X_L_ and Bcl-2 with *K*_D_ values of 36.9 nM and 17.4 nM, respectively, and weak binding affinity for Bak with a *K*_D_ value of 70.0 μM (the green lines in Fig. 5e and Supplementary Fig. S8). Collectively, full-length Bfk that lacks the BC groove remains inert with respect to apoptotic regulation, but upon cleavage by caspases and detachment of the N-terminal fragment, the truncated form of Bfk exhibits its pro-apoptotic characteristics by enabling the BH3 domain to interact with other Bcl-2 family proteins.

## 4 Discussion

Human Bfk, belonging to the Bcl-2 family, is predominantly expressed in tissues of the gastrointestinal tract and its expression is substantially reduced in tumors of the gastrointestinal tract such as the colon, small intestine, and stomach [20], [21]. Additionally, since upregulated Bfk expression promotes apoptosis, human Bfk has been generally regarded as a pro-apoptotic protein [20]. However, the mechanism underlying Bfk-mediated apoptotic regulation has been elusive, given that the unique domain composition of pro-apoptotic Bfk (harboring BH2 and BH3 domains) does not correspond to any of the pro-apoptotic protein subgroups among Bcl-2 family proteins. Indeed, Bfk is neither a pore-former nor a BH3-only protein. Moreover, a three-dimensional structure of Bcl-2 family protein comprising only BH2 and BH3 domains has not been reported to date. In this study, we report the crystal structure of human Bfk, as the first structure among Bcl-2 family proteins containing BH2 and BH3 domains. The overall structure of Bfk exhibited a Bcl-2 fold with high structural similarity to those of multi-domain Bcl-2 family proteins but lacked the BC groove due to the absence of the BH1 domain. Rather, Bfk-specific key residues of helix α5 and the way of forming the core structure in the Bfk structure resembled those of Bid, a BH3-only protein in the Bcl-2 family, suggesting that Bfk exerts pro-apoptotic activity in a manner similar to Bid.

Bid is a Bcl-2 family protein that bridges the intrinsic and extrinsic apoptotic pathways, as tBid translocates to the mitochondrial outer membrane to initiate MOMP, following cleavage of full-length Bid by caspase-3 and caspase-8 in the cytosol [45], [47]. It was revealed that tBid forms an extended structure with a C-shaped configuration and embeds into the mitochondrial outer membrane via helices α5-α6, thereby enabling the efficient recruitment of cytosolic Bax or Bak to the mitochondrial outer membrane for further pore formation [17], [18], [48]. In the Bfk structure, Bfk possesses the helix α5 that shares distinct properties with Bid, which is distinguished from helices α5 constituting the BH1 domain conserved in other multi-domain Bcl-2 family proteins (Fig. 4). In particular, helix α5 of Bfk and Bid contains predominantly aliphatic residues at the center and positively charged residues (Arg110 in Bfk and Lys146 in Bid) in the N-terminal direction (Fig. 4). In addition, the helix α5 in both Bfk and Bid structures is covered by helix α1 containing the pseudo-BH4 domain, of which the invariant residues make hydrophobic contacts between helices α1 and α5 (Fig. 5f,g), implying that they share structural and sequential features in the formation of their distinct folds. It is interesting that tBid utilizes helices α5-α6 to associate with the mitochondrial outer membrane instead of a transmembrane helix that other Bcl-2 family proteins have at C-terminus in many cases [6]. In this regard, tBfk that lacks a transmembrane region but shares a similar motif in the helix α5 with tBid might also adopt its helix α5-α6 region for a subcellular localization and association with a membrane. Therefore, we suggest that Bfk and Bid have the same mechanism underlying the formation of the truncated conformation and association with MOM, in which tBid dissociates from helices α1-α1′ upon caspase cleavage and contact with mitochondrial membranes [49], [50], [51] and its exposed helix α5 embeds into mitochondrial outer membranes [13], [17], [18]. However, the cellular localization of tBfk has not yet been identified and the cell-based evidence for the unfolding of tBfk in the presence of a membrane to elicit a pro-apoptotic signal remains lacking. Therefore, our speculation of the membrane-associated subcellular localization of tBfk to promote apoptosis needs to be addressed through further studies.

Since the truncated Bfk, through cleavage by caspases, becomes a pro-apoptotic protein [23], recognition of the cleavage site on the flexible loop of Bfk by caspases can be one of the rate-limiting steps for further activation. In the Bfk crystal structure, we observed the intermolecular interactions between two adjacent molecules in the crystal structure of monomeric Bfk, especially through the flexible α1′-α2 loop containing a caspase cleavage site. Accordingly, it is assumed that the loop part next to the cleavage site is fixed, so that the caspases can easily recognize the exposed site. Together with MD simulation, the α1′-α2 loop bound to another Bfk molecule was shown to be considerably stable. Such interactions between two Bfk molecules could be an artifact due to crystallization under high concentrations, but since a dimeric form was also observed during the gel filtration step (Supplementary Fig. S9), however minor, it is possible that the loop stabilization through the interplay between two Bfk molecules could play a role in the truncation and activation processes.

tBfk would be structurally rearranged to an active conformation that exposes its BH3 domain to interact with other Bcl-2 family proteins. The comparative MD simulation-based studies on Bfk and Bid showed that the truncated forms from both Bfk and Bid exhibit elevated fluctuations of the BH3-containing helix α2 (Fig. 5c,d), which tend to expose the BH3 domain. Furthermore, as tBfk was reported to exhibit pro-apoptotic activity in a Bax/Bak-dependent manner [23], we demonstrated that the Bfk BH3 domain is capable of binding to Bak (*K*_D_ = 70 μM) (Fig. 5). Therefore, tBfk would be induced to release the BH3-containing helix α2 for further activation of Bax/Bak-mediated apoptosis through conformational changes as shown with tBid. Interestingly, kinetic constants such as on and off rates of Bfk BH3 against Bcl-X_L_, Bcl-2, and Bak in our SPR experiments are shown to be fast compared to other BH3 domains [52], [53], [54]. In our comparative SPR experiments between Bfk BH3 and Bid BH3 against Bcl-2 family proteins, the Bfk BH3 tends to exhibit slightly faster off rates (Supplementary Figs. S8a and S4a). The fast on and off rates of Bfk BH3 might be advantageous for a ‘hit and run’ mechanism that is a possible model for an effector activation [55]. Although whether the direct binding of BH3-only proteins such as Bid activates the pore formation of Bax/Bak into MOM remains debated, many studies have shown that the Bid BH3 domain induces the oligomerization of Bax/Bak essential for further pore formation via transient binding with sub-micromolar affinity [6], [56], [57]. Although our study has shown the direct binding of Bfk BH3 domain to Bak with a fast off rate, it is not enough to fully demonstrate the role of Bfk BH3 as a direct activator because it might act as a sensitizer, like Bad, which elicits pro-apoptotic activity but does not directly activate the pore forming of Bax and/or Bak. Thus, the Bfk BH3 domain needs to be further investigated in the cellular experimental system with respect to whether it could promote MOMP by activating Bcl-2 family pore-formers.

Although Bfk resembles Bid in various aspects, the BH2 domain at the C-terminus of Bfk does not exist in Bid. In a previous study on Bcl-G, another Bcl-2 family protein harboring BH2 and BH3 domains, the autorepression of pro-apoptotic signaling was suggested as a function of the BH2 domain because the pro-apoptotic activity of Bcl-G following deletion of the BH2 domain was increased [58]. Structural analysis of Bfk in comparison with the BH3-only Bid or multi-domain Bcl-2 family proteins revealed that the BH2 domain on the C-terminal helices α7–α8 of Bfk establishes interaction networks with helix α5 in the Bfk core region, contributing to the structural integrity of the core Bcl-2 fold (Fig. 4f). Indeed, MD simulations with tBfk and tBid with and without the BH2 domain, respectively, showed that the BH2-containing helices α6–α8 of tBfk were less flexible than those of tBid (Fig. 5c,d). Thus, the BH2 domain of Bfk is thought to attenuate the structural rearrangement when the Bcl-2 core fold is wound, leading to a delay in the rapid exposure of the BH3 domain and thus reducing pro-apoptotic signals. This is supported by the increased apoptosis observed upon deletion of the BH2 domain from Bcl-G [58], although its concomitant molecular basis could not be verified due to a lack of structural knowledge of Bcl-G. The presence of the BH2 domain in Bfk appears to strictly regulate its pro-apoptotic activity compared to that with the BH3-only protein Bid. Human Bfk is mainly expressed in the gastrointestinal tract, where stem cells undergo rapid proliferation, differentiation to terminal mature cells, and cell death by apoptosis [59]. This suggests a reasonable speculation that Bfk, mainly expressed in gastrointestinal tract, induces apoptosis under stringent regulation through the BH2 domain.

Recently, AlphaFold is emerging as the most powerful tool to predict a protein structure based on the sequence. When we compared the crystal structure of Bfk with the AlphaFold-predicted structure available in the EBI database, despite the high similarity (a Cα root-mean-square deviation value of 0.741) in their overall structures, there are structural differences between them, which might be related to functional aspects. Most importantly, the sidechain of Arg110 on helix α5 in the crystal structure makes a hydrogen bond with Gln155 of helix α7, whereas the one of Arg110 in the AlphaFold-predicted structure is directed toward helix α2 by forming a hydrogen bond with Gln63 (Supplementary Fig. S10). In the crystal structure, the hydrogen bonds of Arg110 with Gln155 and Glu162, are thought to enhance an interaction network of α5 with helices α7–α8, which belong to a region for BH2 domain (Supplementary Fig. S10). Therefore, the conformation of Arg110 seen in the crystal structure that might affect the structural integrity is noteworthy. Another noticeable difference between the crystal structure and the AlphaFold structure is the helix α1′ next to helix α1 (Supplementary Fig. S10). All Bcl-2 family proteins including Bfk have the long loop of the flexible nature between helices α1 and α2, so that most Bcl-2 family proteins and even the structure predicted by AlphaFold present this region as a loop or as in not modeled. Interestingly, Bfk and Bid contain additional helix α1′ before a flexible loop starts, and the first crystal structure of Bfk confirms the existence of such helix α1′ and reveals its difficult-to-predict characteristic structure in detail. Considering that the region for helix α1′ is encompassed by several helices of symmetrical molecules and exhibits a large RMSF during MD simulation, the possibility that the helix α1′ could be caused by crystal contact cannot be excluded, but it might be valuable to compare them and further describe the additional information that can only be obtained through experimental structural determination.

In conclusion, whereas functional mechanisms of Bcl-2 family proteins in the intrinsic pathway of apoptosis have been actively studied, a molecular basis for Bcl-2 family proteins that belong to an orphan member, owing to its unique composition of BH2 and BH3 domains, has been remarkably in veil. In this study, we first reported the crystal structure of human Bfk as an orphan member among the Bcl-2 family proteins, which revealed that the BC groove is absent and its P1-P4 pockets remains blocked by the unique conformation of helices α2-α4. In addition, we opened the possibility of a Bid-like apoptotic mechanism for Bfk via structural, biophysical, and computational analyses. Our findings will provide insight into the molecular basis underlying apoptotic regulation by Bfk as a novel Bcl-2 family protein.

## Funding

This work was supported by the Tumor Microenvironment Global Core Research Center (grant no. 2011-0030001) (B.W.H.) and the Basic Science Research Programs (grant no. NRF-2019R1A2C1090251 and NRF-2020R1C1C1009512) (B.W.H. and H.S.K., respectively) funded by the National Research Foundation (NRF) of the Ministry of Science and ICT of Korea. This work was also supported by the Brain Korea (BK21) PLUS program to the College of Pharmacy at the Seoul National University. H.S.K. was also supported by the National Cancer Center grant of Korea (NCC-2210730 and NCC-2210390).

## CRediT authorship contribution statement

**Dong Man Jang:** Conceptualization, Investigation, Data curation, Writing – original draft, Writing – review & editing, Visualization. **Eun Kyung Oh:** Conceptualization, Investigation, Data curation, Writing – original draft. **Hyunggu Hahn:** Data curation. **Hyoun Sook Kim:** Conceptualization, Writing – review & editing, Supervision, Funding acquisition. **Byung Woo Han:** Conceptualization, Writing – review & editing, Supervision, Funding acquisition.

## Declaration of Competing Interest

The authors declare that they have no known competing financial interests or personal relationships that could have appeared to influence the work reported in this paper.
