# Supplementary material for: Structural insights into apoptotic regulation of human Bfk as a novel Bcl-2 family member
Source: Comput Struct Biotechnol J. 2022 Jan 28;20:745–56. doi: 10.1016/j.csbj.2022.01.023 (PMC8814693; doi:10.1016/j.csbj.2022.01.023)
Supplement: Supplementary data 1 [file mmc1.pdf]

Structural Insight into Apoptotic Regulation of Human Bfk as a Novel Bcl-2 Family Member

Dong Man Jang[1,2,†], Eun Kyung Oh[1,†], Hyunggu Hahn[1], Hyun-Jung Kim[3], Hyoun Sook Kim[2,]*, and Byung Woo Han[1,]*

[1] Research Institute of Pharmaceutical Sciences, College of Pharmacy, Seoul National University, Seoul 08826, Korea

[2] Research Institute, National Cancer Center, Goyang, Gyeonggi 10408, Korea

[†] These authors contributed equally to this work.

* Correspondences: hskim@ncc.re.kr (H.S.K.) and bwhan@snu.ac.kr (B.W.H.); Tel.: +82-2-920-2275 (H.S.K.) and +82-2-880-7898 (B.W.H.)

Supplementary Data

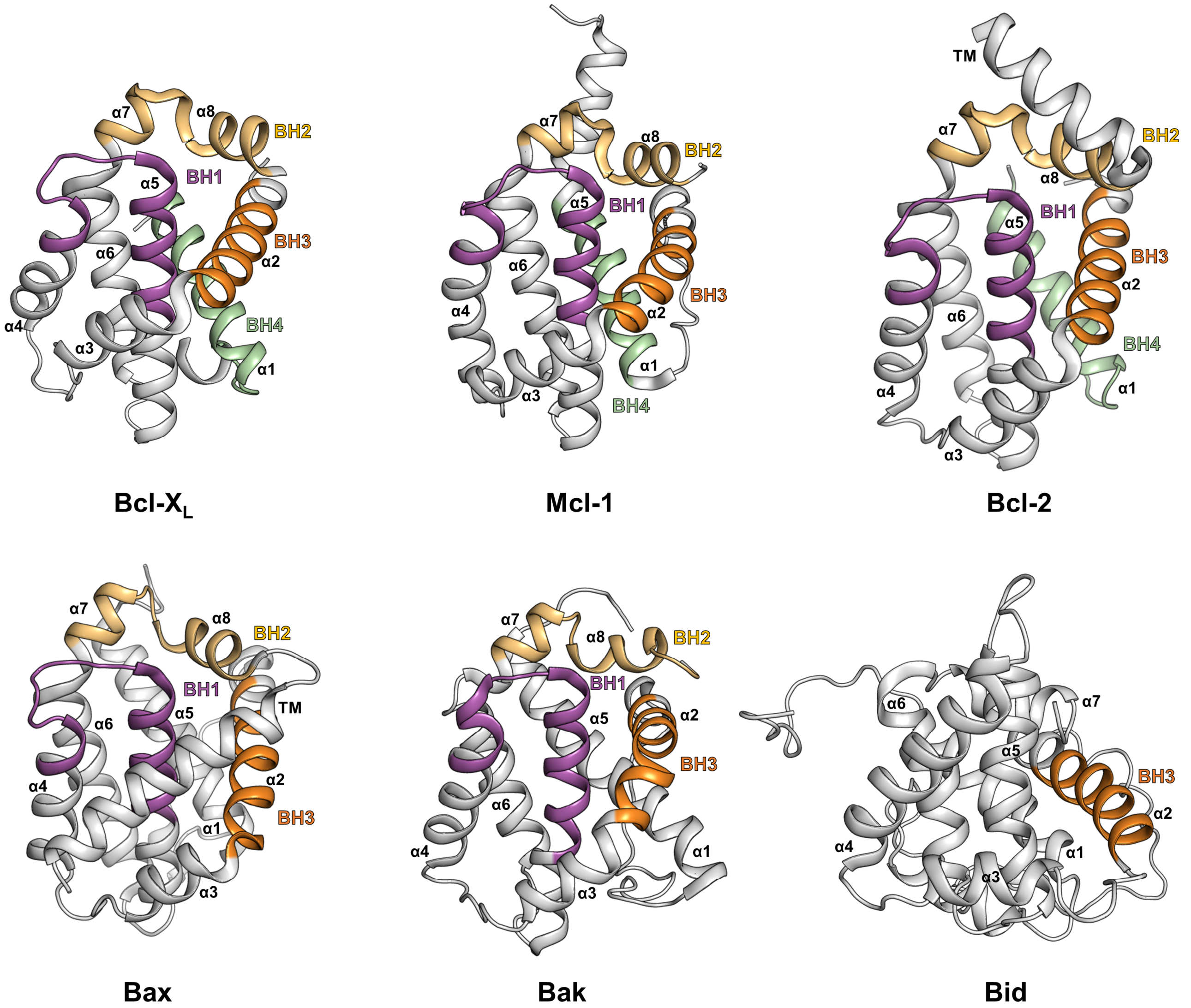

Figure S1. Overall structures of Bcl-2 family proteins. The structures of Bcl-X_L (PDB ID: 6RNU), Mcl-1 (6QGD), Bcl-2 (6QGH), Bax (5W60), Bak (2M5B), and Bid (2BID) are displayed as cartoon representations colored in white in the same view as in Fig. 2a,b. Regions for BH1, BH2, BH3, and BH4 domains are colored in magenta, yellow, orange, and green, respectively. The bound molecules such as inhibitors in the original coordinates are omitted in the representation.

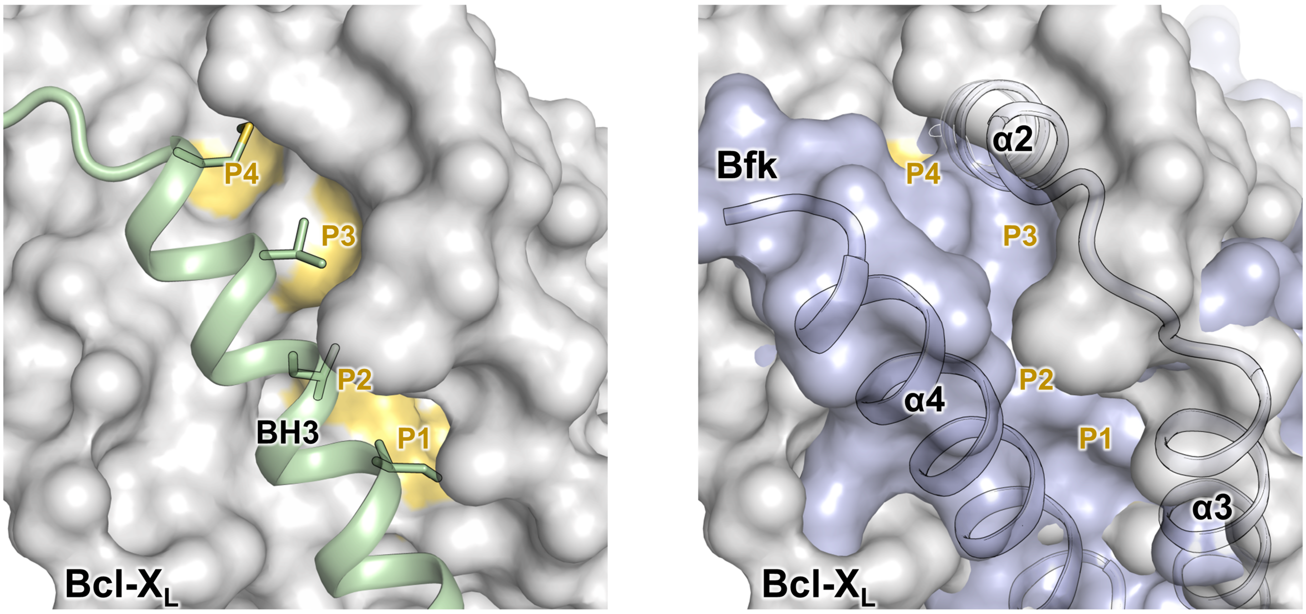

Figure S2. The structural comparison of BC groove between Bfk and Bcl-X$_L$. (Left) The structure of Bcl-X$_L$ in complex with Bid BH3 peptide (PDB code: 4QVE). The structure of Bcl-X$_L$ is shown as a surface representation colored in white. The structure of Bid BH3 peptide is shown as a cartoon representation colored in green. The hydrophobic residues (green-colored stick) of Bid BH3 peptide are binding to the P1–4 pockets (yellow-colored surface area) on the BC groove. **(Right)** The superposition of Bcl-X$_L$ and Bfk. The structure position and representation of Bcl-X$_L$ are identical with (Left) figure except the Bid BH3 peptide that has been removed. The structure of Bfk is shown as a surface representation colored in light blue. The helices α2–α4 of Bfk are drawn with a cartoon representation using a black line.

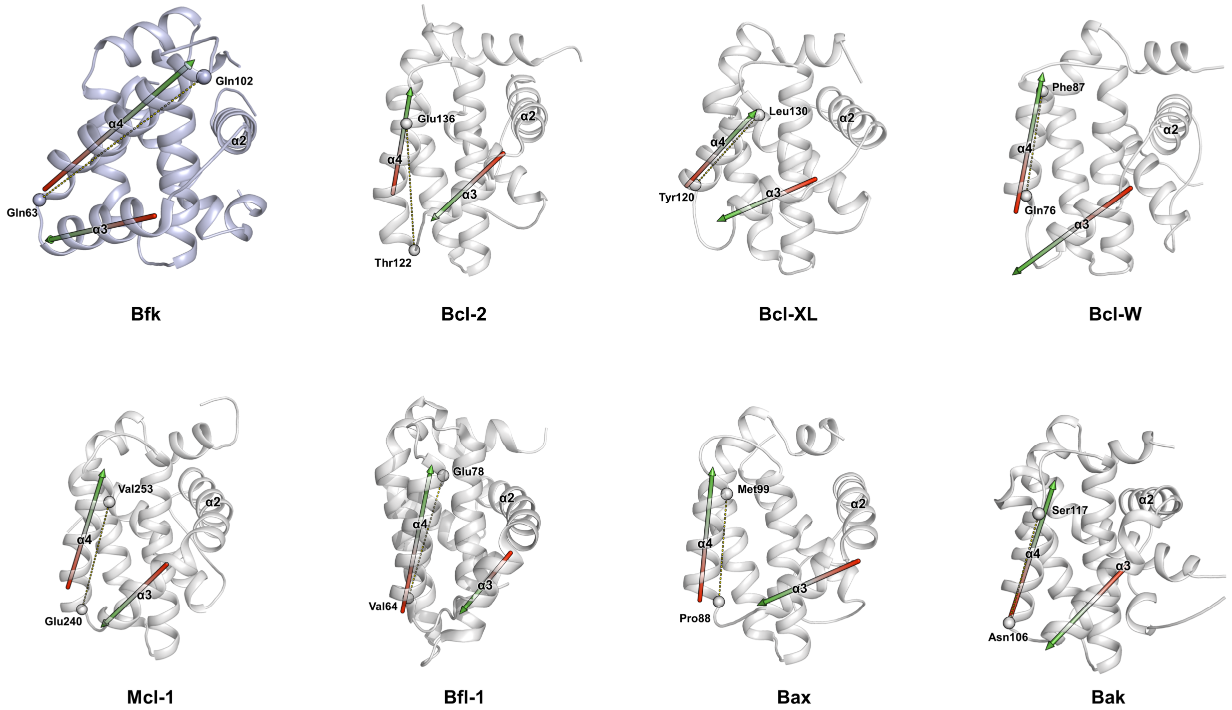

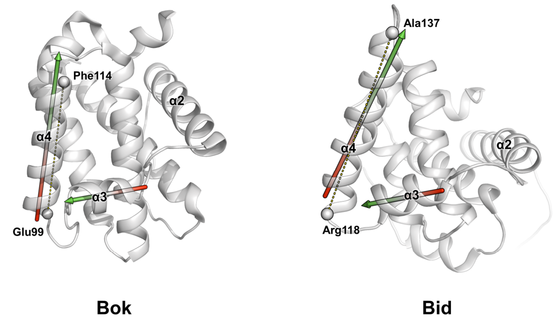

	α3-α4 angle (x°)	Residue range for α3	Residue range for α4
**Bfk**	19.6	E69–K81	Q83–A101
**Bcl-2**	35.6	G110–Q118	G128–R139
**Bcl-X_L**	32.4	A104–H113	Q121–L130
**Bcl-W**	40.4	R59–P72	A75–Q88
**Mcl-1**	41.3	A227–L235	V243–S255
**Bfl-1**	34.7	K53–V59	S63–E78
**Bax**	56.8	N73–A82	P88–S101
**Bak**	37.4	S91–Q101	N106–F119
**Bok**	86.1	N87–L92	R100–A116
**Bid**	34.7	V106–R114	R118–A137

Figure S3. The length of helix α4 and angle between helices α3–α4 of Bcl-2 family proteins. The structures of Bcl-2 (PDB ID: 1G5M), Bcl-X_L (1MAZ), Bcl-W (1O0L), Mcl-1 (2MHS), Bfl-1 (5WHI), Bax (1F16), Bak (2YV6), Bok (6CKV), and Bid (2BID) are used for a calculation. Overall structures are shown as cartoon representations and Cα atoms for measuring the length of helix α4 are indicated as spheres labeled with corresponding residues. Distances between spheres for helix α4 are drawn with colored-dotted lines. The angles between helices α3–α4 are measured by a PyMOL plugin, AngleBetweenHelices. The helices for measuring angles are indicated as green and red colored arrows. Helices α3–α4 angle and residue range to define helix α3 and helix α4 are summarized as the table.

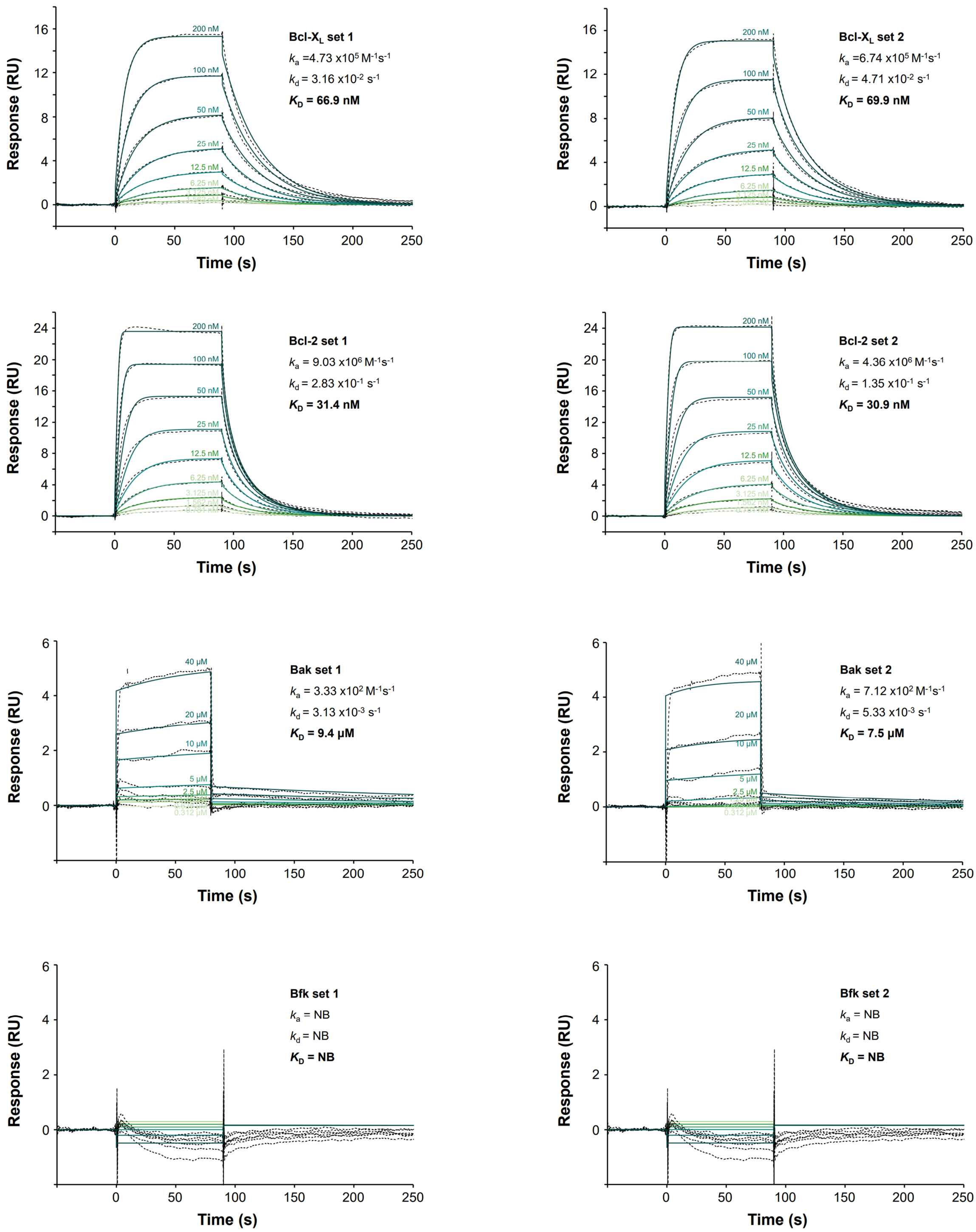

Figure S4 (related to Figure 2E). Affinity analysis of a representative BH3 peptide against Bfk and multi-domain Bcl-2 family proteins by a surface plasmon resonance method. SPR sensorgrams show binding of Bcl-X_L, Bcl-2, Bak, and Bfk to the immobilized BH3 peptide derived from Bid (EDIIRNIARHLAQVGDSMDRS) at increasing concentrations (0.781, 1.562, 3.125, 6.25, 12.5, 25, 50, 100, and 200 nM for Bcl-X_L and Bcl-2, and 0.31, 0.62, 1.25, 2.5, 5, 10, 20, and 40 μM for Bak and Bfk). K_D values shown on the right side are calculated by fitting (colored lines) the responses (dotted lines).

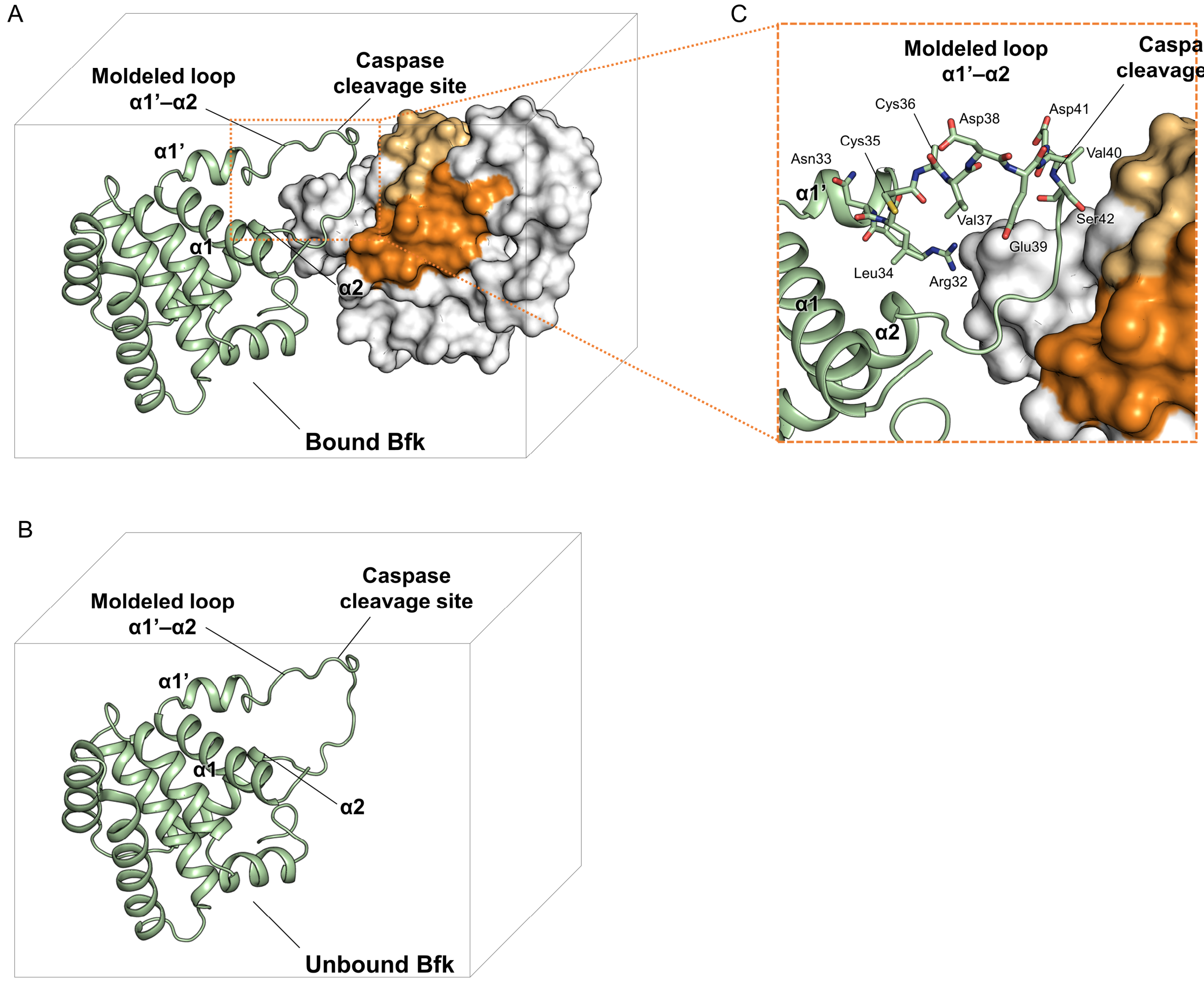

Figure S5. Orthorhombic unit systems for molecular dynamics (MD) simulations. Repeating unit cells in the periodic boundary conditions of MD simulations for crystallographic dimer Bfk (A) and monomer Bfk (B). One molecule colored in green (A,B) is displayed as a cartoon representation. Invisible residues in the loop connecting helix α1’ and helix α2 are modeled using Prime in Schrödinger2021-2. The other molecule (A) is displayed as a surface representation colored in yellow for BH2-containing region, orange for BH3-containing region, and white for the others. (C) A close-up view of the interface of two Bfk molecules. The modeled residues in the loop connecting helix α1’ and helix α2 are displayed as stick representations. The caspase cleavage site in the modeled loop is indicated. Oxygen, nitrogen, and sulfur atoms are colored in red, blue, and yellow, respectively.

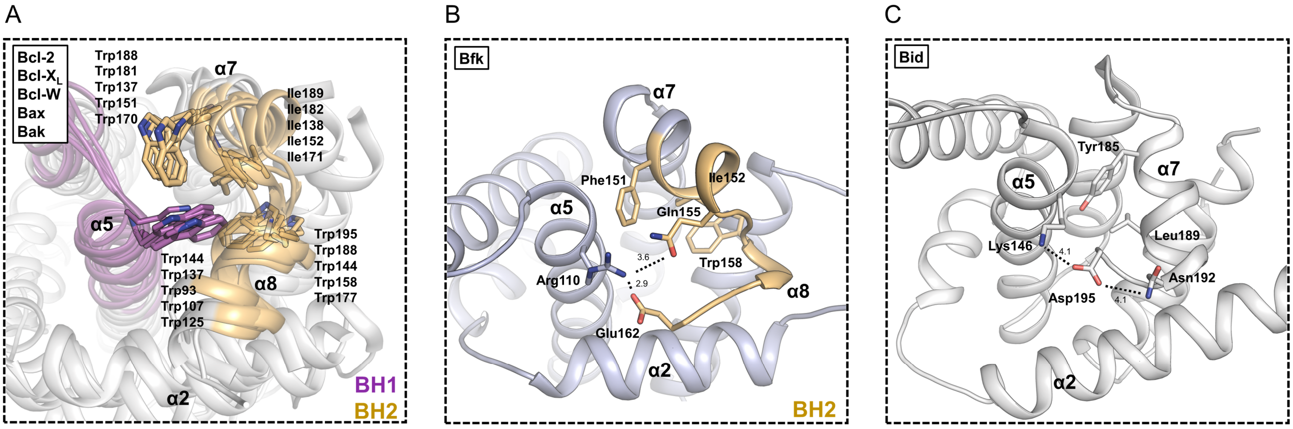

Figure S6. Comparison of interaction networks between helix α5 and helices α7–α8 of Bcl-2 family proteins. (A) A close-up view of multi-domain Bcl-2 family proteins in the same view as in Fig. 4f,g. The structures of Bcl-2, Bcl-X_L, Bcl-W, Bax, and Bak are superimposed. Close-up views of Bfk (B) and Bid (C). The secondary structures and interacting residues are displayed as cartoon and stick representations, respectively. BH1 and BH2 domain are colored in magenta and yellow, respectively. Hydrophilic intearctions including hydrogen bonds are indicated as dotted lines with the distances.

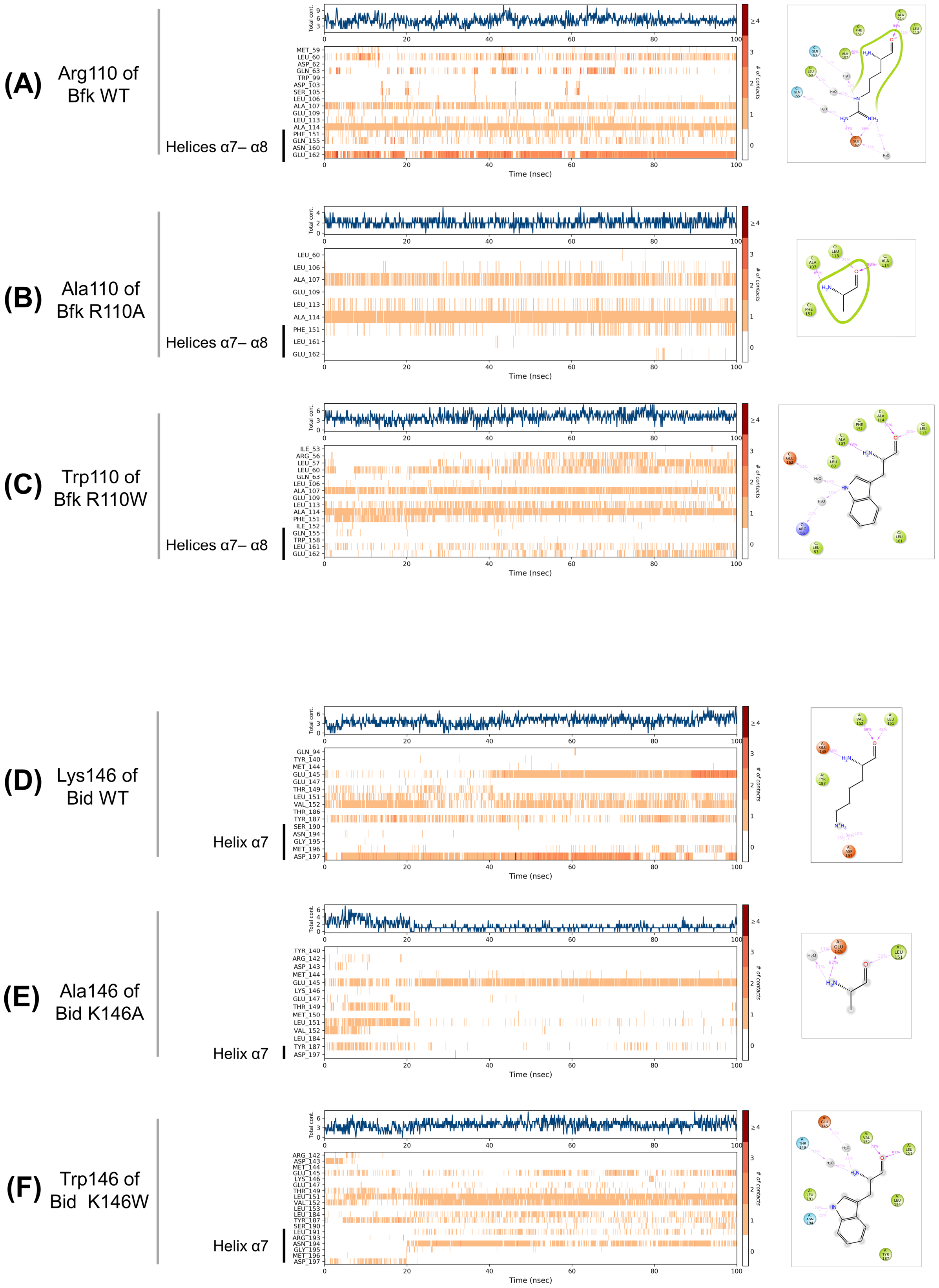

Figure S7. Comparison of interaction networks between Bfk and Bid over 100 ns MD simulation. The mutant models of Bfk R110A, Bfk R110W, Bid R146A, and Bid R146W were generated from the wild-type structures of Bfk and Bid using Schrödinger suite 2021-2. **(A–C)** The timeline representations indicate the number of contacts between Bfk Arg110 **(A)** or Bfk Ala110 **(B)** or Bfk

Trp110 **(C)** and interacting residues in the Bfk structure as orange lines over 100 ns. **(D–F)** The timeline representations indicate the number of contacts between Bid Lys146 **(D)** or Bid Ala146 **(E)** or Bid Trp146 **(F)** and interacting residues in the Bid structure as orange lines over 100 ns. The contacts include hydrogen bonds, hydrophobic contacts, ionic interaction, and water bridges. The regions for helices α7–α8 are indicated with black line. The schematic diagram shows interactions between key residue and its interacting protein residues. The spheres colored in orange, green, and light blue indicate the negative charged, hydrophobic, and polar residues, respectively. The grey colored spheres indicate water.

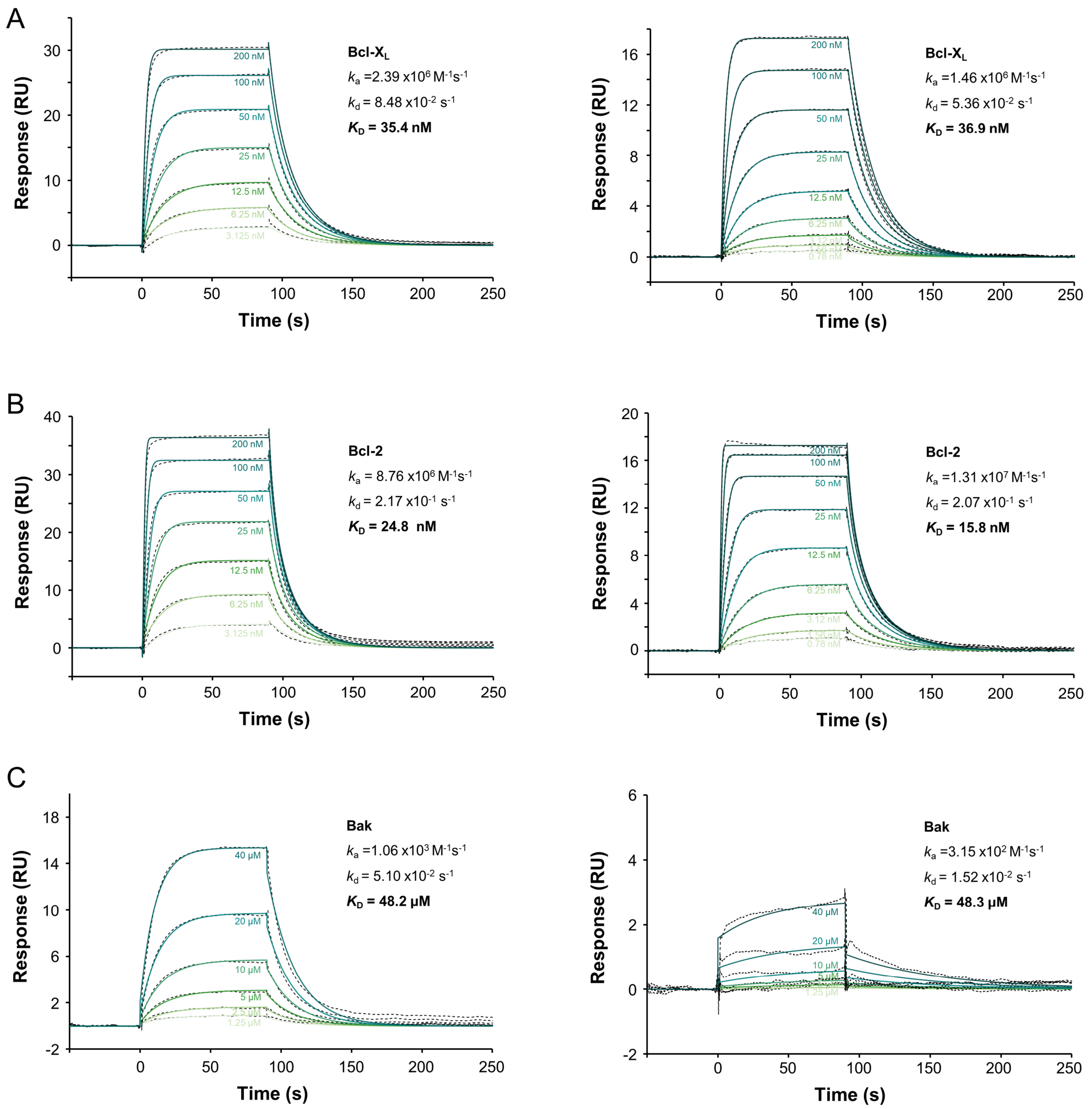

Figure S8 (related to Figure 5E). Surface plasmon resonance (SPR) analyses of binding affinities for the Bfk BH3 peptide with Bcl-X$_L$, Bcl-2, and Bak. SPR sensorgrams show binding of Bcl-X$_L$, Bcl-2, and Bak to the immobilized Bfk BH3 peptide at increasing concentrations (3.125, 6.25, 12.5, 25, 50, 100, and 200 nM for Bcl-X$_L$ and Bcl-2, and 1.25, 2.5, 5, 10, 20, and 40 μM for Bak). K_D values shown on the right side are calculated by fitting (colored lines) the responses (dotted lines).

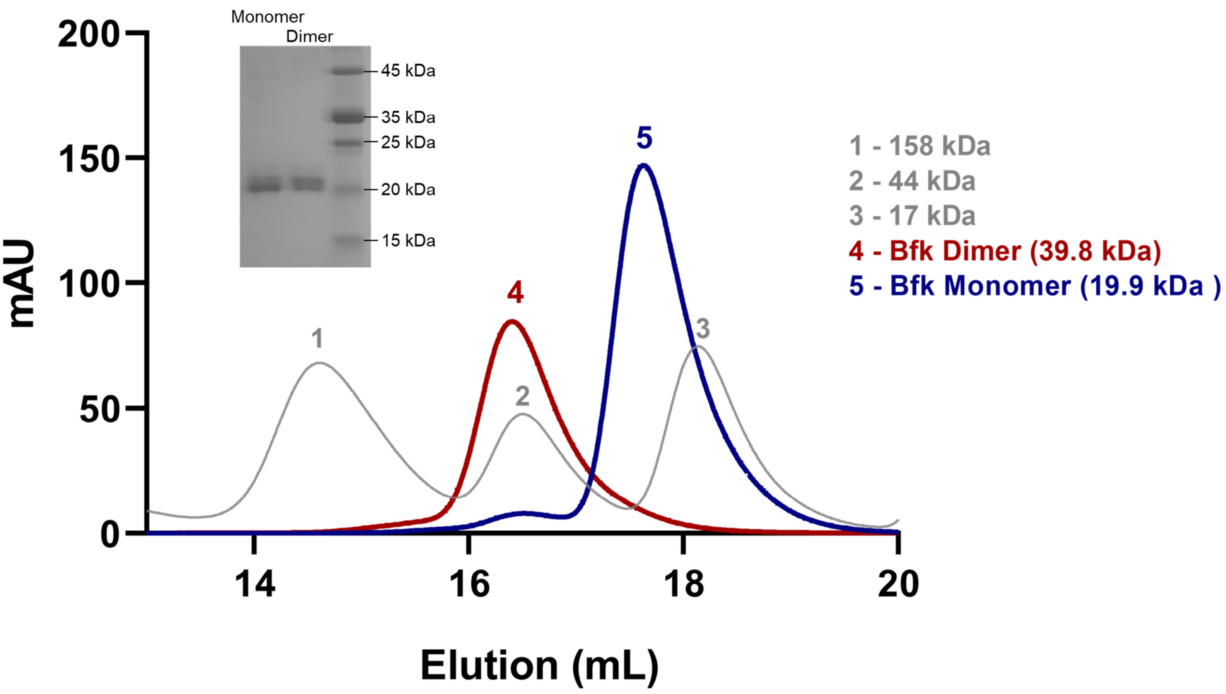

Figure S9. The oligomeric states of Bfk proteins in solution. Superdex 200 increase 10/300 GL column (GE Healthcare, Chicago, IL, USA) was used to analyze the oligomeric status of two Bfk forms that were each isolated in the previous step. The monomeric and dimeric forms of Bfk proteins are drawn with blue- and red-colored lines, respectively. Gel filtration standard samples are drawn with a grey-colored line. The theoretical molecular weights for standard samples (1–3), Bfk dimer (4), and Bfk monomer (5) are written on the right side. The SDS-PAGE analysis for the elution fraction is shown beside the chromatogram.

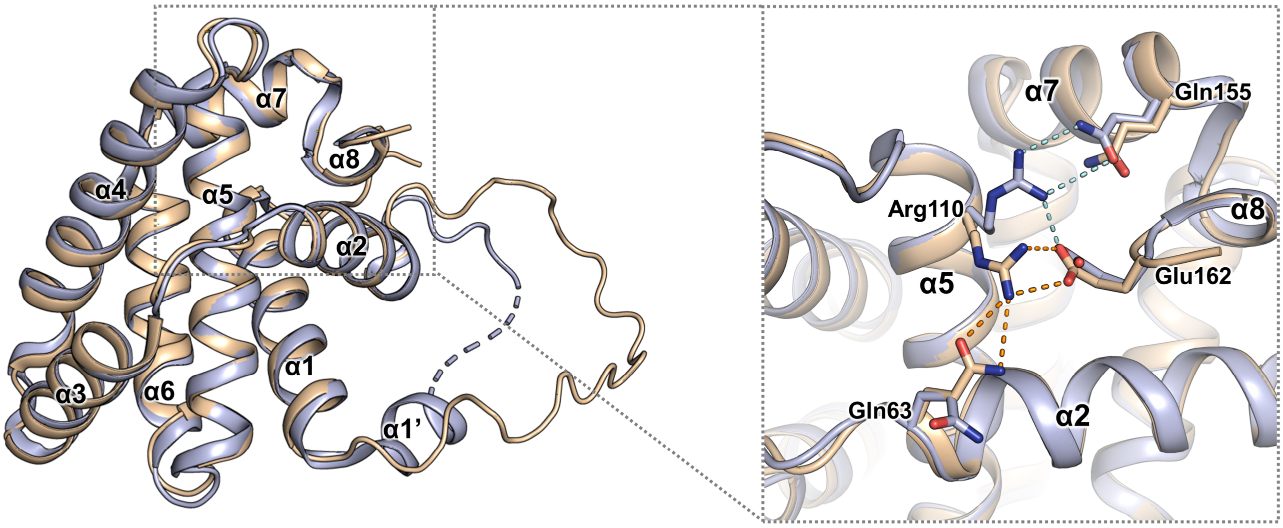

Figure S10. Structural comparison between the crystal structure and the AlphaFold-predicted structure of Bfk. (Left) Superposition of the crystal structure (light blue) and the AlphaFold-predicted structure (wheat) of Bfk. **(Right)** Close-up view of interaction networks among helices α2, α5, and α7–α8. Interacting residues are shown as stick representations. Hydrogen bonds are depicted with dotted lines (colored in cyan and orange for the crystal structure and AlphaFold-predicted structures, respectively).
